# Provision of specific dental procedures by general dentists in the National Dental Practice-Based Research Network: questionnaire findings

**DOI:** 10.1186/1472-6831-15-11

**Published:** 2015-01-22

**Authors:** Gregg H Gilbert, Valeria V Gordan, James J Korelitz, Jeffrey L Fellows, Cyril Meyerowitz, Thomas W Oates, D Brad Rindal, Randall J Gregory, National Dental PBRN Collaborative Group

**Affiliations:** Department of Clinical and Community Sciences, School of Dentistry, University of Alabama at Birmingham, SDB Room 109, 1530 Third Avenue South, Birmingham, AL 35294-0007 USA; Department of Restorative Dental Sciences, College of Dentistry, University of Florida, Gainesville, FL USA; Westat, Rockville, MD USA; Kaiser Permanente Center for Health Research, Portland, OR USA; Eastman Institute for Oral Health, Rochester, NY USA; Department of Periodontics, School of Dentistry, University of Texas Health Science Center at SanAntonio, San Antonio, TX USA; HealthPartners Institute for Education and Research, Minneapolis, MN USA; General Dentist, Winston-Salem Dental Care, Winston-Salem, NC USA

**Keywords:** Dentist practice patterns, Practice-based research, Dentistry, Health services research

## Abstract

**Background:**

Objectives were to: (1) determine whether and how often general dentists (GDs) provide specific dental procedures; and (2) test the hypothesis that provision is associated with key dentist, practice, and patient characteristics.

**Methods:**

GDs (n = 2,367) in the United States National Dental Practice-Based Research Network completed an Enrollment Questionnaire that included: (1) dentist; (2) practice; and (3) patient characteristics, and how commonly they provide each of 10 dental procedures. We determined how commonly procedures were provided and tested the hypothesis that provision was substantively related to the three sets of characteristics.

**Results:**

Two procedure categories were classified as “uncommon” (orthodontics, periodontal surgery), three were “common” (molar endodontics; implants; non-surgical periodontics), and five were “very common” (restorative; esthetic procedures; extractions; removable prosthetics; non-molar endodontics). Dentist, practice, and patient characteristics were substantively related to procedure provision; several characteristics seemed to have pervasive effects, such as dentist gender, training after dental school, full-time/part-time status, private practice vs. institutional practice, presence of a specialist in the same practice, and insurance status of patients.

**Conclusions:**

As a group, GDs provide a comprehensive range of procedures. However, provision by individual dentists is substantively related to certain dentist, practice, and patient characteristics. A large number and broad range of factors seem to influence which procedures GDs provide. This may have implications for how GDs respond to the ever-changing landscape of dental care utilization, patient population demography, scope of practice, delivery models and GDs’ evolving role in primary care.

**Electronic supplementary material:**

The online version of this article (doi:10.1186/1472-6831-15-11) contains supplementary material, which is available to authorized users.

## Background

The field of dentistry is undergoing substantial changes that are relevant to the range of services that general dentists (GDs) provide. These include the changing picture of dental economics and dental care utilization, demography of patient populations, the scope of practice, changing delivery models, access to care, and an increased interest in the relationship between oral health and general health [1, 2]. One vision for the future of general dentistry is that it serves as a segue into the health care system at large, offering an opportunity to monitor, refer, or treat both oral health and general medical conditions [3]. In that vision, GDs function in a primary care role, and the comprehensiveness of the procedures that GDs provide takes on additional significance.

GDs assume a dual role as providers of primary oral health care and as gatekeepers who refer patients to specialists [4]. In 1996, about 81% of all dental visits in the United States were provided by GDs [5]. Nonetheless, a study of Michigan children’s Medicaid claims data found that about 20% of dentists only provided diagnostic and preventive services for their Medicaid patients, meaning that for these children Medicaid coverage was not a segue into comprehensive primary dental care that included restorative care [6]. Most dental extractions are provided by GDs, especially among lower-income patients [7]. Most non-surgical periodontal care is provided in GDs’ offices, and increases in demand for periodontal services are being met mainly by GDs, not periodontists [5, 8]. As long as they are capable of providing the service to the standard of care, GDs substitute for a specialist’s care, instead of complementing it, which has economic and delivery system implications [5].

Decisions made to treat or refer may be a means for GDs to adapt to changing economic demand and the needs of their patient population. It is known that characteristics of the patient population that the GD serves can substantially influence the specific types of services provided [9]. An Australian study led to the conclusion that dental service rates are influenced by a large number of small effects from a wide range of dentist, practice, and patient factors [10]. A few studies have identified certain dentist characteristics as being associated with provision of endodontic, periodontal, and oral surgical services [11–14]. However, little is known about how commonly GDs provide directly a comprehensive range of procedures, or about factors associated with this provision. Therefore, our objectives were to: (1) determine whether and how often GDs provide specific dental procedures; and (2) test the hypothesis that provision is significantly associated with key dentist, practice, and patient population characteristics.

## Methods

The large number of GDs in the United States National Dental Practice-Based Research Network provides an opportunity to better understand the range of services that GDs provide. The network is a consortium of dental practices and dental organizations focused on improving the scientific basis for clinical decision-making [15]. It is committed to maximizing the practicality of conducting research in everyday clinical practice across geographically dispersed regions and diverse practice types. The network has a wide representation of practice types, treatment philosophies, and patient populations, including diversity regarding race, ethnicity, gender, geography and rural/urban area of residence of both its practitioners and their patients. Many details about the network are available at its web site (http://nationaldentalpbrn.org).

Practitioners comprise a convenience sample that is recruited at dental meetings, continuing education courses, by other network practitioners, announcements from dental organizations, mass mailings using dental licensure lists, or via the network’s internet site. As part of the enrollment process, practitioners complete an Enrollment Questionnaire that describes characteristics about themselves, their practice(s), and their patient population. These characteristics are listed in Table 1 and are the characteristics tested in this article for their association with provision of specific dental services. The categories of dental service types are also listed in Table 1. Questionnaire items, which have documented test/re-test reliability, were taken from our previous work in a practice-based study of dental care and a PBRN that ultimately led to the network [16, 17]. The typical enrollee completes the questionnaire online, although a paper option is available. This study was approved by the University of Alabama at Birmingham Institutional Review Board and participants provided informed consent.

**Table 1:** **Characteristics and dental procedure categories**

A. Characteristics tested for their association with provision of specific dental procedures		
Dentist characteristics	Practice characteristics	Patient population characteristics
Gender	Number of different practice locations at which you practice	Age composition of patients
Age	Practice location (rural/urban)	Hispanic/Latino ethnicity composition of patients
Hispanic/Latino ethnicity	Type of main practice	Race composition of patients
Race	Dental specialists at same practice location	Insurance coverage of patients
Year of graduation from dental school	How long a patient has to wait for appointments	Regularity of dental visitation by patients
Training after dental school		
Membership in dental organizations		
Full-time/part-time^a^		
**B. Dental procedure categories**		
1	Non-implant restorative (amalgams, composites, crowns, veneers, bridges, posts, foundations, etc.)	
2	Esthetic procedures (procedures done for esthetic reasons only; composites, crowns, veneers, etc.)	
3	Extractions (surgical and non-surgical)	
4	Removable prosthetics (full and partial dentures)	
5	Endodontic therapy- anteriors/premolars	
6	Endodontic therapy- molars	
7	Implants (prosthetic and surgical procedures for implants)	
8	Periodontal therapy- non-surgical (includes scaling/root planning that you do personally)	
9	Orthodontic treatment	
10	Periodontal therapy- surgical	

^a^Part-time was defined as working less than 32 hours each week in patient care.

### Statistical methods

The outcomes of interest were how commonly (not at all; occasionally; routinely) each of the 10 specific dental procedures listed in Table 1 were provided by the GD. Dentist, practice, and patient population characteristics functioned as independent variables. For characteristics that were categorical (e.g., dentist gender), two-way frequency tables (with counts and percentages) were created and statistical tests were done using Fisher’s exact test. When the characteristic was numerical (e.g., dentist age), one-way analysis of variance (with means and standard deviations) was done.

The large sample size and multiple statistical comparisons create a circumstance in which associations between dental characteristics and procedure provision can be statistically significant even when the magnitude of the differences is quite small. In contrast to highlighting associations that are statistically significant (even if corrections were made for multiple comparisons), we chose to highlight those that we would label “substantively related”. For the sake of identifying specific criteria before analyses were begun, “substantively related” was defined as occurring when the association between the dentist/practice characteristic and the procedure type is p ≤ 0.001 and: 1) for categorical variables, the percentage difference between any of the comparison groups (e.g., male dentists compared to female dentists) is at least 10% (for example, 15% vs. 25%) for any of “never”, “occasionally”, or “routinely” categories; 2) for non-categorical variables, the mean value across the three “never”, “occasionally”, or “routinely” categories demonstrates a consistent increase or decrease across categories. Multivariable models of the dental procedures as a function of dentist, practice, and patient population characteristics were generated using multinomial logistic regression (producing separate models for the outcomes “occasionally” and “routinely”, using “never” as the reference category) that accounted for possible confounding by other covariates. All analyses were conducted using SAS version 9.3 [SAS Institute, Inc., Cary, North Carolina, USA].

## Results

As of October 31, 2013, a total of 4,641 persons had enrolled, of whom 2,993 were dentists (2,367 GDs; 615 dental specialists; 11 not specified), 1,239 were dental hygienists, and the remainder were in some other category, such as dental students or non-practitioners. The 2,367 GDs who had enrolled as of October 31, 2013 are the subject of this report; the earliest enrollment date possible was April 15, 2012. The characteristics of the GDs in this sample are available [see Additional file 1]. Table 2 shows how commonly 10 dental procedures are provided by GDs, in ascending order of providing them, from “not at all” to “routinely”. Based on the percentage of GDs who reported providing these procedures either occasionally or routinely, we categorized these procedures into three groups: “very common” (more than 75%); “common” (50%-75%); “uncommon” (less than 50%). Two to five procedure types comprised each of these categories.

**Table 2:** **Percent (number) of procedures provided by GDs, in ascending order of providing them “not at all”**

Procedure type	Not at All	Occasionally	Routinely	Total
“very common” procedures				
Non-implant restorative	2% (49)	2% (41)	96% (2233)	100% (2323)
Esthetic procedures	5% (109)	36% (835)	59% (1384)	100% (2328)
Extractions	5% (119)	31% (726)	64% (1473)	100% (2318)
Removable prosthetics	6% (130)	38% (876)	57% (1316)	100% (2322)
Endodontic therapy – anteriors/premolars	16% (376)	26% (605)	58% (1339)	100% (2320)
“common” procedures				
Endodontic therapy – molars	38% (883)	26% (603)	36% (831)	100% (2317)
Implants	39% (889)	39% (902)	22% (514)	100% (2305)
Periodontal therapy – non-surgical	40% (917)	37% (859)	23% (541)	100% (2317)
“uncommon” procedures				
Orthodontic treatment	68% (1565)	23% (532)	9% (217)	100% (2314)
Periodontal therapy - surgical	69% (1578)	26% (601)	6% (127)	100% (2306)

Table 3 identifies the associations between practice characteristics and service provision that were “substantively related”, using the criteria described in the Statistical Methods section. Detailed full tables of results of these associations are available [see Additional file 2]).

**Table 3:** **Summary of whether association between the characteristic and frequency of providing the service is “substantively related”** ^**a**^

Characteristic of the dentist, practice, or patient population	Non-implant restorative	Esthetic procedures	Extractions	Removable Prosthetics	Endodontic-anteriors/premolars	Endodontic- molars	Implants	Periodontal-non-surgical	Orthodontic	Periodontal-surgical
Dentist gender		Males do more		Males do more	Males do more	Males do more	Males do more			Males do more
Dentist age in years			Older dentists do less							Older dentists do more
Dentist Hispanic/Latino ethnicity										
Dentist race			Minority dentists do more				Minority dentists do less	Minority dentists do more		
Year of graduation from dental school			Less-recent graduates do less							Less-recent graduates do more
Training after dental school…										
No formal training program							No training, do less		No training, do less	No training, do less
Completed an AEGD program										
Completed a GPR program										
I am a FAGD		FAGD do more				FAGD do more	FAGD do more		FAGD do more	FAGD do more
Completed MAGD							MAGD do more			MAGD do more
Completed some other training program							Other training, do more		Other training, do more	Other training, do more
Member of…										
American Dental Association							ADA members do more			
Academy of General Dentistry		AGD members do more					AGD members do more		AGD members do more	
Hours in practice in patient contact ^b^		FT do more	FT do more	FT do more	FT do more	FT do more	FT do more	FT do more	FT do more	FT do more
Number of different locations at which you see patients	More locations, do less	More locations, do less	More locations, do more							
Practice location			Inner city and rural do more	Inner city and rural do more			Non-inner city and suburban do more			
Type of main practice (full)	Non-academic, do more	Public funded or HMO do less	Non-academic do more	Public funded do less	Public funded do less	Public funded do less	Private and academic do more		Private do more	
Type of main practice (private)	Private do more	Private do more	Private do less	Private do more	Private do more	Private do more	Private do more		Private do more	
Whether your practice has at the same location a …										
Not applicable (only GDs at this location)		If only GD there, do more		If only GD there, do more			If only GD there, do more	If only GD there, do more		If only GD there, do more
Endodontist					If endo there, do less	If endo there, do less		If endo there, do less		If endo there, do less
Oral & Maxillofacial Surgeon	If oral surg there, do less		If oral surg there, do less	If oral surg there, do less remov	If oral surg there, do less endo				If oral surg there, do less	
Orthodontist								If ortho here, do less perio	If ortho here, do less ortho	
Pediatric dentist		If pediatric there, do less		If pediatric there, do less			If pediatric there, do less	If pediatric there, do less		
Periodontist								If perio there, do less		
Prosthodontist				If pros there, do less						
Other	If Other there, do less	If Other there, do less	If Other there, do less	If Other there, do less			If Other there, do less			
How long a patient has to wait …										
for a new patient exam appt.		Less wait, do more				Less wait, do more	Less wait, do more		Less wait, do more	
for a treatment procedure appt.		Less wait, do more				Less wait, do more	Less wait, do more			
in the waiting room	Less wait, do more	Less wait, do more					Less wait, do more			
Percentage of patients who are…										
1-18 years old		Younger patients, do less		Younger patients, do less			Younger patients, do less			
19-44 years old										
45-64 years old		Older patients, do more		Older patients, do more			Older patients, do more			
65 or older				Older patients, do more			Older patients, do more			
Percentage Hispanic patients	More Hispanic, do less	More Hispanic, do less		More Hispanic, do more			More Hispanic, do less	More Hispanic, do more		
Percentage of patients whose race is…										
White/Caucasian		More white, do more	More white, do less				More white, do more	More White, do less		
Black/African-American			More Black/AA, do more				More Black/AA, do less			
American Indian/Alaska Native										
Asian										
Native Hawaiian/Pacific Islander										
Other		More Other, do less		More Other, do less			More Other, do less			
Percentage of patients who are…										
Covered by a private insurance program	More pvt ins, do more	More pvt ins, do more			More pvt ins, do more	More pvt ins, do more				
Covered by a public program		More public ins, do less					More pub ins, do less			
Not covered by any third party and pays out of pocket		More out-of-pocket, do more	More out-of-pocket, do less	More out-of-pocket, do more			More out-of-pocket, do more		More out-of-pocket, do more	More out-of-pocket, do more
Receiving free care or substantially reduced fees courtesy of this practice		More free care, do less				More free care, do less	More free care, do less			
Percent of patients who come…										
For one visit only	More 1 visitors, do less	More 1 visitors, do less					More 1 visitors, do less			
Occasionally only when they have an emergency or specific problem		More occ visitors, do less					More occ visitors, do less			
Irregularly whether or not they have a problem			More irreg., do more							
Regularly as recommended or whether or not they have a problem	More regular visitors, do more	More regular visitors, do more					More regular visitors, do more			

^a^“Substantively related” is defined as occurring when the association between the dentist/practice characteristic and the procedure type is p ≤ 0.001 and: (1) for categorical variables, the percentage difference between any of the comparison groups (e.g., male dentists compared to female dentists) is at least 10% (i.e., 10 percentage points) for any of “never”, “occasionally”, or “routinely” categories; (2) for non-categorical variables, the mean value across the three “never”, “occasionally”, or “routinely” categories demonstrates a consistent increase or decrease across categories.

^b^FT: full-time.

As shown in Table 3, several dentist characteristics were substantively related to provision of procedures. Because the effect of dentist gender could be confounded by year of graduation and full-time/part-time status, regression analyses were done to account for all three characteristics simultaneously. After accounting for these two potential confounders, males were still more likely to perform endodontic procedures (anteriors/premolars and molars), implants, and surgical periodontal therapy, but gender was no longer substantively related to esthetic and removable prosthetic procedures.

The effect of dentist race could be confounded by the racial distribution and insurance status of the dentist’s patient population. Regression analyses were done to account for all three characteristics simultaneously. After this adjustment, there was no association between dentist race and dental procedures.

As shown in Table 3, full-time/part-time status was substantively related to all but one of the procedure types. These effects remained even once dentist gender was taken into account during regression analyses.

The type of main practice was substantively related to most of the procedure types. Because these effects could be confounded by insurance status of the patient population, regression analyses were done to account for practice type and insurance status. Practice type remained substantively related to the dental procedures shown in Table 3, even after accounting for insurance status.

As shown in Table 3, several patient population characteristics were substantively related to provision of specific procedures; namely, age group, ethnic and racial distributions, insurance coverage, and visitation behavior. Regression analyses were done to account for race, insurance status, and visitation behavior simultaneously. The associations noted in Table 3 remained, except that the percentage of Black/African-American patients in a practice was no longer substantively related to extractions and implants, and visitation behavior was no longer substantively related to implants.

## Discussion

### Provision rates for specific services

Orthodontics and surgical periodontal therapy were uncommon procedures; more than two-thirds of GDs reported that they do not do these procedures. For comparison, the literature provides very little information about what percentage of GDs does any orthodontic services, but more information is available about how commonly GDs provide surgical periodontal therapy. A 2007 study of GDs in Virginia [13] observed that about 24% reported doing surgical pocket reduction, comparable to the 32% of GDs in our study who do surgical periodontal therapy of any type. A total of 15% of dentists in Victoria, Australia provided periodontal surgery [18]. Doing more or less periodontal surgery may be a means for GDs to adjust services to the demand for services overall in their practices.

As expected, a substantially higher percentage of GDs reported doing non-surgical periodontal therapy as compared to surgical periodontal therapy. The 60% who do non-surgical periodontal therapy in our study is similar to the 57% figure among dentists who were members of the Michigan Dental Association [19]. The majority of GDs in our study (62%) reported doing at least some molar endodontics, and 84% do at least some anterior or premolar endodontics. These percentages are similar when judged by a different measure available from dentists in Washington State, the percentage of endodontic care that is done by endodontists: 26% of anterior root canal treatment was done by endodontists, 29% of premolars and 50% of molars [20]. Similar to the circumstance with periodontal surgery, it is possible that provision of endodontic services is a means for GDs to adjust to the availability of dental specialists and to overall demand for services in their practices.

Our study queried how often dental implant services are provided, regardless of whether these procedures are surgical or prosthetic; 61% of GDs reporting doing at least some implant procedures. The few other studies available asked about the surgical placement of implants, without regard to the prosthetic procedure. For example, 16% of Virginia GDs reported performing the surgical component of implant placement in a 2007 survey [13], and in a study of Boston University dental school graduates, only 10% of GDs place implants [12]. Studies ultimately may emerge that evaluate across a full range of procedures the long-term effectiveness of procedures done by GDs as compared to specialists, or effectiveness studies of procedures done by GDs who do a large volume of these procedures as compared to GDs who do not do a large volume; such studies may ultimately affect the percentage of GDs who choose to do these procedures.

### Association between dentist characteristics and service provision

#### Dentist gender

Several dentist characteristics were substantively related to provision of specific services. Dentist gender was substantively related to six of the 10 service types; males were more likely to provide each of these service types. The salience of dentist gender is an emerging finding across several treatment considerations in the network, even once gender differences in dental school graduation year and full-time/part-time status have been taken into account. We hypothesized that dentist gender would not be significantly related to service provision once graduation year and full-time status was taken into account. Consequently, it is noteworthy that four of the six gender/service associations remained substantively related to service provision in multiple regressions when graduation year and full-time status were taken into account. This does seem to be a recurring theme in most of the literature to date: although an analysis of dental claims data in Washington State revealed no gender differences in practice patterns [21], other studies have observed that male dentists are more likely to provide specific procedures. These include studies of Nova Scotia and Virginia GDs in which male dentists were more likely to perform non-surgical periodontal therapy [8, 14], endodontic services [11], and dental extractions [12, 22].

#### Dentist race

White dentists reported doing more implant procedures and less extractions. However, when adjusted in multiple regressions for their patient population’s racial distribution and insurance coverage, the association with dentist race was no longer substantively related to service provision.

#### Dentist year of graduation

Less-recent graduates in the network do substantively less extractions, but more periodontal surgery. When adjusted in multiple regressions for their patient population’s insurance coverage, the association with dentist graduation year remained substantively related to service provision. The literature is mixed in its conclusion about the effect of graduation year. Periodontal referrals have been associated with year of dental school graduation [13, 18]. Endodontic referrals were less likely among GDs with 6–10 years of experience, compared to those with more than 10 years of experience [11].

#### Advanced training by the GD

Whether or not the dentist had some type of formal training after dental school graduation was substantively related to service provision, but conclusions depended upon the type of training. When the comparison was ‘no training’ versus ‘training of any type’, those with no training were substantively less likely to provide implant, surgical periodontal, and orthodontic services. However, when the analysis took the next step to incorporate more detail about this training, those who had Fellowship in the Academy of General Dentistry (FAGD) or Mastership in the Academy of General Dentistry (MAGD) status or those who had completed some other type of training that did not include an Advanced Education in General Dentistry (AEGD) or General Practice Residency (GPR) program, were substantively more likely to provide specific service types; AEGD or GPR training was not substantively related to any service provision. The reasons for these differences were not queried in this study, but it is possible that AEGD and GPR training increases experience with many procedure types at the expense of depth with a small number of procedures, while FAGD, MAGD, or other type of training provides greater depth of experience with a small number of procedures, which is reflected in being more likely to provide these specific services. A comparable pattern was observed for dentists who are members of the American Dental Association (ADA) or Academy of General Dentistry. In a study of Virginia GDs, those with more advanced training, such as AEGD training, were more likely to refer for implant placement instead of providing these services directly [13]. In a study of AEGD and GPR training in the United States, dentists with postgraduate training were more likely to provide periodontal surgery and implants, but differences in other services were not statistically significant, except for multi-unit fixed bridges in which GDs with advanced training were actually less likely to provide these services [22].

#### Full-time status

We hypothesized that full-time dentists would be more likely to provide a wider range of service types. Whether the dentist was in employment on a full-time basis was substantively related to 9 of the 10 service types; full-time dentists were more likely to provide each of these services when compared to part-time dentists. We are not aware of other reports in the literature about this phenomenon, but this could be a salient consideration when making workforce projections.

### Association between practice characteristics and service provision

#### Number of practice locations

Dentists who provide care at more than one location reported doing more dental extractions and less restorative care (including esthetically-focused restorative care). We are not aware of other reports in the literature about this phenomenon. When adjusted in multiple regressions for their patient population’s insurance coverage, the association with number of practice locations remained substantively related to service provision.

#### Practice location

Inner city and rural practices were more likely to provide extraction and removable prosthodontic procedures, while suburban and urban practices not in the inner city were more likely to provide implants. The literature does not provide any information to which to compare, except regarding distance from a periodontist and referrals. Farther distances from a periodontist have been associated with GDs being more likely to refer periodontal care among Virginia dentists [14], but farther distances were associated with less periodontal referrals among British dentists [23], members of the Michigan Dental Association [19], and Nova Scotia dentists [8]. It is not clear if GDs’ provision of periodontal services is confounded by the GDs’ emphasis on periodontal diagnosis in the practice; that is, the extent to which periodontal diagnosis is emphasized may affect the number of patients for whom a periodontal procedure or referral is even considered.

#### Type of main practice

Private practices, as compared to clinics with large public sources of funding, are more likely to provide “higher-end” services, such as restorative care and esthetically-focused restorative care, endodontics, implants, and orthodontics. This finding also parallels certain patient population characteristics listed later in Table 3 (insurance coverage and dental visitation behavior).

#### Dental specialists on site

We hypothesized that when the GD was in a practice setting without any specialist on site, then the GD would be more likely to provide a wider range of services. We also hypothesized that if a specialist was available on site, then the GD would be less likely to provide services that could be done by that specialist. As evident in Table 3, both hypotheses were true. A limitation of this study is that we do not know if the practice employs dental hygienists. This could be a factor in how commonly certain procedures are provided, such as non-surgical periodontal care, although the directions of effect in the literature are conflicting [14, 23, 24].

#### Waiting times

We hypothesized that the shorter the wait times for new patient examinations, treatment procedures, and in the waiting room, then the more likely that the GD would be to report providing a wider range of services. As evident in Table 3, these hypotheses were true.

### Association between patient population characteristics and service provision

Evident in Table 3, service provision was substantively related to patient age group, Hispanic/Latino ethnicity, and race, in expected directions. When adjusted in regression analyses for their patient population’s insurance coverage, age, ethnicity, and race were no longer substantively related to service provision.

#### Insurance coverage and visitation behavior

GDs were more likely to provide restorative care, esthetically-focused restorative care, and endodontics when their patient population had a higher percentage of private insurance coverage, and correspondingly lower-end service types when their patient population had higher percentages with public insurance or those who rely on free or reduced-fee care. Although the literature has a large amount of information about the role of insurance coverage on patients’ demand for care, little has been reported about the role of insurance coverage and visitation behaviors on whether GDs provide a full range of service types. A study of Michigan dentists did report that socioeconomic status and insurance status affected whether GDs refer their periodontal patients to a periodontist [19]. As dental insurance changes or expands to other patient populations in the future, GDs may adjust their mix of services to adapt to this changing demand. Other patient population characteristics were significantly associated with provision of specific services. It is possible that behavioral measures or disease prevalence of the patients in the practice would be a more-direct and stronger influence on referral or provision, so this would be a worthy avenue for future research in this area, if such measures could be obtained feasibly.

This study does have certain limitations that should be kept in mind when making inferences from it. We collected data via self-reports that were not validated by other means, such as observational data or dental records abstraction. As with all self-reported data, it is possible that participants’ responses may not reflect their actual behavior. Also, the study questionnaire queried how commonly specific procedures are done, but for the sake of brevity we did not query the converse: how commonly procedures are referred to specialists. It is possible that these responses would have yielded different results. Additionally, network members are not recruited randomly, so factors associated with network participation (e.g., an interest in clinical research) may make network dentists unrepresentative of dentists at large. While we cannot assert that network dentists are entirely representative, we can state that they have much in common with dentists at large, while also offering substantial diversity in these characteristics. This assertion is warranted because: 1) substantial percentages of network GDs are represented in the various response categories of the characteristics listed in Table 1 [15]; 2) findings from several network studies document that network GDs report patterns of diagnosis and treatment that are similar to patterns determined from non-network GDs [25–28]; and 3) the similarity of network GDs to non-network GDs using the best available national source, the 2010 ADA Survey of Dental Practice [29]. Regarding similarities to dentists in the ADA survey, the ADA survey samples both ADA members and non-ADA members, is based on a national probability sample, and provides the most comprehensive information on the characteristics of United States dentists. However, the ADA sample is limited to dentists in private practice, is based on a 29% response rate, and provides results from 2009. Because ADA Survey respondents cannot be practitioners in public health clinics, federal or tribal facilities, community health centers, or dental schools (provided they do not see private patients in the dental school), comparisons to network practitioners are limited in that regard. A total of 79% of ADA survey participants were general dentists, compared to 80% of network practitioners. Gender distribution in the ADA survey was 17% female [p. 6], compared to 27% female for network practitioners. The mean age in the ADA survey was 52.8 years [p. 5]; compared to 49.9 years for network practitioners. ADA Survey practices with one dentist accounted for 78% of practices [p. 8], compared to 81% for network dentists. A total of 64% of patients had private dental insurance in the ADA Survey [p. 23], compared to 59% for network practitioners.

## Conclusions

This study adds to the very limited literature about how commonly GDs provide directly a comprehensive range of procedures, and factors associated with this provision. As a group, GDs do indeed provide a comprehensive range of procedures. However, provision by individual dentists is significantly associated with certain dentist, practice, and patient characteristics. Although the study design precludes direct cause-and-effect conclusions, we infer that effects from a large number and broad range of factors seem to influence which procedures GDs provide in their practices. These findings may have implications for how GDs respond to the changing picture of dental economics and dental care utilization, demography of patient populations, the scope of practice, changing delivery models, access to care, and their evolving role in primary care.

## Electronic supplementary material

Additional file 1: **Characteristics of enrolled practitioners, by practice type. Bivariate cross-tabulations.** (PDF 209 KB)

Additional file 2: **Frequency of provision of procedure types, by dentist, practice, and patient characteristics. Bivariate cross-tabulations.** (PDF 959 KB)

## Abbreviations

ADA: American Dental Association
AEGD: Advanced Education in General Dentistry (residency program)
FAGD: Fellowship in the Academy of General Dentistry
GD: General dentist
GPR: General Practice Residency
MAGD: Mastership in the Academy of General Dentistry.
