# Supplementary material for: Provision of specific dental procedures by general dentists in the National Dental Practice-Based Research Network: questionnaire findings
Source: BMC Oral Health. 2015 Jan 22;15:11. doi: 10.1186/1472-6831-15-11 (PMC4324862; doi:10.1186/1472-6831-15-11)
Supplement: Supplementary file 1 — Additional file 1: Characteristics of enrolled practitioners, by practice type. Bivariate cross-tabulations. (PDF 209 KB) [file 12903_2014_496_MOESM1_ESM.pdf]

## Additional file 1. Characteristics of enrolled practitioners, by practice type (as of January 25, 2013) [unabridged version]

| | | Dentists | | | | | Hygienists |
|---|---|---|---|---|---|---|---|
| | | Private vs. Non-Private | | | General vs. Specialist | | |
| Characteristic | | All | Private Practice | Non-Private | General Practitioner | Specialist | All |
| | | | | | | | |
| Overall | | 1427 | 954 | 135 | 897 | 193 | 443 |
| | | | | | | | |
| Number of different locations at which you see patients | | | | | | | |
| | 1 | 1160 (81.4%) | 801 (84.0%) | 92 (68.1%) | 774 (86.3%) | 120 (62.2%) | 311 (70.2%) |
| | 2 | 195 (13.7%) | 118 (12.4%) | 30 (22.2%) | 96 (10.7%) | 52 (26.9%) | 85 (19.2%) |
| | 3 | 43 ( 3.0%) | 22 ( 2.3%) | 6 ( 4.4%) | 16 ( 1.8%) | 12 ( 6.2%) | 15 ( 3.4%) |
| | More than 3 | 27 ( 1.9%) | 13 ( 1.4%) | 7 ( 5.2%) | 11 ( 1.2%) | 9 ( 4.7%) | 32 ( 7.2%) |
| | Total | 1425 (100.0%) | 954 (100.0%) | 135 (100.0%) | 897 (100.0%) | 193 (100.0%) | 443 (100.0%) |
| | Missing/Blank | 2 | 0 | 0 | 0 | 0 | 0 |
| Gender | | | | | | | |
| | Male | 1026 (72.7%) | 697 (73.8%) | 79 (59.8%) | 637 (71.6%) | 143 (76.1%) | 10 ( 2.3%) |
| | Female | 385 (27.3%) | 247 (26.2%) | 53 (40.2%) | 253 (28.4%) | 45 (23.9%) | 432 (97.7%) |
| | Total | 1411 (100.0%) | 944 (100.0%) | 132 (100.0%) | 890 (100.0%) | 188 (100.0%) | 442 (100.0%) |
| | Missing/Blank | 16 | 10 | 3 | 7 | 5 | 1 |
| Mean age | | | | | | | |
| | Mean (S.D.) | 50 (11.7) | 50.3 (11.3) | 48.5 (12.3) | 49.7 (11.5) | 51.8 (10.6) | 43.6 (11.1) |
| Hispanic/Latino | | | | | | | |
| | Yes | 71 ( 5.0%) | 39 ( 4.1%) | 19 (14.4%) | 38 ( 4.3%) | 20 (10.5%) | 47 (10.7%) |
| | No | 1338 (95.0%) | 902 (95.9%) | 113 (85.6%) | 847 (95.7%) | 170 (89.5%) | 393 (89.3%) |
| | Total | 1409 (100.0%) | 941 (100.0%) | 132 (100.0%) | 885 (100.0%) | 190 (100.0%) | 440 (100.0%) |
| | Missing/Blank | 18 | 13 | 3 | 12 | 3 | 3 |
| Race | | | | | | | |
| | White/Caucasian | 1175 (83.6%) | 792 (84.0%) | 100 (76.9%) | 730 (82.5%) | 164 (86.3%) | 392 (89.1%) |
| | Black/African-American | 51 ( 3.6%) | 33 ( 3.5%) | 7 ( 5.4%) | 36 ( 4.1%) | 5 ( 2.6%) | 20 ( 4.5%) |

**Additional file 1. Characteristics of enrolled practitioners, by practice type (as of January 25, 2013) [unabridged version]**

| | | Dentists | | | | | Hygienists |
|---|---|---|---|---|---|---|---|
| | | Private vs. Non-Private | | | General vs. Specialist | | |
| Characteristic | | All | Private Practice | Non-Private | General Practitioner | Specialist | All |
| | American Indian/Alaska Native | 4 ( 0.3%) | 4 ( 0.4%) | 0 ( 0.0%) | 4 ( 0.5%) | 0 ( 0.0%) | 2 ( 0.5%) |
| | Asian | 133 ( 9.5%) | 82 ( 8.7%) | 21 (16.2%) | 85 ( 9.6%) | 17 ( 8.9%) | 13 ( 3.0%) |
| | Native Hawaiian/Pacific Islander | 1 ( 0.1%) | 1 ( 0.1%) | 0 ( 0.0%) | 1 ( 0.1%) | 0 ( 0.0%) | 2 ( 0.5%) |
| | Other | 42 ( 3.0%) | 31 ( 3.3%) | 2 ( 1.5%) | 29 ( 3.3%) | 4 ( 2.1%) | 11 ( 2.5%) |
| | Total | 1406 (100.0%) | 943 (100.0%) | 130 (100.0%) | 885 (100.0%) | 190 (100.0%) | 440 (100.0%) |
| | Missing/Blank | 21 | 11 | 5 | 12 | 3 | 3 |
| Practice location | | | | | | | |
| | Inner City of Urban Area | 193 (13.6%) | 81 ( 8.5%) | 46 (34.3%) | 100 (11.2%) | 28 (14.6%) | 97 (22.0%) |
| | Urban (not inner city) | 424 (29.9%) | 285 (30.0%) | 49 (36.6%) | 266 (29.7%) | 66 (34.4%) | 101 (23.0%) |
| | Suburban | 602 (42.4%) | 466 (49.0%) | 13 ( 9.7%) | 393 (43.9%) | 89 (46.4%) | 181 (41.1%) |
| | Rural | 200 (14.1%) | 119 (12.5%) | 26 (19.4%) | 136 (15.2%) | 9 ( 4.7%) | 61 (13.9%) |
| | Total | 1419 (100.0%) | 951 (100.0%) | 134 (100.0%) | 895 (100.0%) | 192 (100.0%) | 440 (100.0%) |
| | Missing/Blank | 8 | 3 | 1 | 2 | 1 | 3 |
| Hours in practice | | | | | | | |
| | 32 or more hours | 1137 (80.8%) | 815 (86.4%) | 69 (52.3%) | 750 (84.7%) | 136 (70.8%) | 280 (63.8%) |
| | Less than 32 hours | 271 (19.2%) | 128 (13.6%) | 63 (47.7%) | 135 (15.3%) | 56 (29.2%) | 159 (36.2%) |
| | Total | 1408 (100.0%) | 943 (100.0%) | 132 (100.0%) | 885 (100.0%) | 192 (100.0%) | 439 (100.0%) |
| | Missing/Blank | 19 | 11 | 3 | 12 | 1 | 4 |
| How long a patient has to wait Mean (S.D.) | | | | | | | |
| | For a new patient exam appt. (days) | 8.1 (13.3) | 6.8 (9.7) | 19.5 (29.5) | 7.9 (14.0) | 10.3 (15.5) | 12.1 (45.3) |
| | For a treatment procedure appt. (days) | 8.2 (13.5) | 7.1 (9.2) | 17.3 (31.2) | 7.9 (14.5) | 10.1 (12.2) | 8.5 (21.0) |
| | In the waiting room (mins) | 8.2 ( 8.2) | 7.6 (8.4) | 11.7 ( 9.8) | 8.0 ( 8.9) | 8.4 ( 7.2) | 8.8 ( 9.1) |

**Additional file 1. Characteristics of enrolled practitioners, by practice type (as of January 25, 2013) [unabridged version]**

| | | Dentists | | | | | Hygienists |
|---|---|---|---|---|---|---|---|
| | | Private vs. Non-Private | | | General vs. Specialist | | |
| Characteristic | | All | Private Practice | Non-Private | General Practitioner | Specialist | All |
| Percentage of patients who are Mean (S.D.) | | | | | | | |
| | 1-18 years old | 22.6 (23.7) | 22.6 (22.5) | 23.7 (30.0) | 18.3 (15.2) | 43.4 (39.4) | 19.8 (19.6) |
| | 19-44 years old | 27.6 (12.7) | 27.2 (11.2) | 28.7 (17.0) | 28.8 (11.2) | 20.9 (14.0) | 28.2 (13.4) |
| | 45-64 years old | 32.9 (13.3) | 32.9 (12.2) | 33.8 (17.5) | 33.8 (11.3) | 28.6 (19.4) | 32.7 (12.9) |
| | 65 or older | 20.1 (12.1) | 19.8 (11.0) | 20.9 (16.2) | 20.1 (11.1) | 18.3 (14.9) | 21.8 (14.5) |
| Percentage of patients who are of Hispanic/Latino ethnicity Mean (S.D.) | | | | | | | |
| | Hispanic | 15.3 (18.4) | 14.4 (17.1) | 28.1 (27.4) | 15.4 (19.2) | 18.1 (18.5) | 20.1 (19.4) |
| Percentage of patients whose race is Mean (S.D.) | | | | | | | |
| | White/Caucasian | 66.4 (22.8) | 68.0 (21.8) | 55.2 (26.5) | 66.7 (23.2) | 65.7 (20.2) | 64.0 (23.1) |
| | Black/African-American | 16.8 (16.2) | 16.1 (15.8) | 20.3 (19.6) | 16.9 (16.9) | 15.1 (13.6) | 16.4 (14.6) |
| | American Indian/Alaska Native | 2.9 ( 9.5) | 1.8 ( 3.3) | 9.3 (24.2) | 2.7 ( 9.1) | 2.6 ( 9.6) | 4.1 (10.0) |
| | Asian | 8.5 ( 9.3) | 8.8 ( 9.6) | 9.1 ( 8.6) | 8.3 ( 8.9) | 10.8 (11.5) | 7.7 ( 8.9) |
| | Native Hawaiian/Pacific Islander | 1.4 ( 2.9) | 1.4 ( 2.7) | 1.9 ( 4.0) | 1.4 ( 2.8) | 1.5 ( 3.4) | 2.2 ( 5.6) |
| | Other | 15.3 (21.4) | 14.2 (19.9) | 27.2 (30.7) | 15.3 (21.9) | 17.8 (21.5) | 15.5 (18.2) |
| Percentage of patients who are Mean (S.D.) | | | | | | | |
| | Covered by a private insurance program | 60.7 (24.2) | 63.9 (20.9) | 41.8 (33.0) | 61.3 (23.4) | 61.9 (24.6) | 58.8 (29.1) |
| | Covered by a public program | 17.7 (24.1) | 14.2 (19.4) | 37.0 (34.2) | 16.6 (23.0) | 18.4 (23.5) | 18.6 (27.0) |
| | Not covered by any third party and pays out of pocket | 22.7 (17.2) | 22.9 (16.4) | 18.1 (19.6) | 22.5 (16.6) | 21.8 (17.8) | 20.3 (18.9) |

**Additional file 1. Characteristics of enrolled practitioners, by practice type (as of January 25, 2013) [unabridged version]**

| | | Dentists | | | | | Hygienists |
|---|---|---|---|---|---|---|---|
| | | Private vs. Non-Private | | | General vs. Specialist | | |
| Characteristic | | All | Private Practice | Non-Private | General Practitioner | Specialist | All |
| | Receiving free care or substantially reduced fees courtesy of this practice | 5.1 (12.7) | 3.0 ( 4.4) | 19.6 (29.8) | 5.3 (13.2) | 3.6 ( 6.1) | 8.9 (21.3) |
| Percent patients who come Mean (S.D.) | | | | | | | |
| | For one visit only | 10.2 (14.4) | 9.2 (13.0) | 16.3 (18.9) | 8.6 ( 9.4) | 17.2 (25.7) | 8.7 (11.2) |
| | Occasionally only when they have an emergency or specific problem | 13.0 (11.5) | 12.5 (10.8) | 16.9 (14.1) | 13.1 ( 9.1) | 12.3 (18.8) | 11.4 ( 9.4) |
| | Irregularly whether or not they have a problem | 15.9 (10.9) | 15.6 ( 9.8) | 18.9 (15.2) | 16.7 (10.1) | 12.2 (12.7) | 15.7 (10.9) |
| | Regularly as recommended or whether or not they have a problem | 63.3 (20.5) | 64.7 (18.7) | 54.4 (25.3) | 62.6 (18.5) | 68.0 (25.2) | 64.9 (19.2) |
| Internet access in practice | | | | | | | |
| | Access for administrative staff | 1332 (93.3%) | 891 (93.4%) | 126 (93.3%) | 838 (93.4%) | 182 (94.3%) | 400 (90.3%) |
| | Access in the operatories | 890 (62.4%) | 587 (61.5%) | 93 (68.9%) | 554 (61.8%) | 126 (65.3%) | 290 (65.5%) |
| | Access for clinical staff outside operatories | 1162 (81.4%) | 777 (81.4%) | 111 (82.2%) | 732 (81.6%) | 158 (81.9%) | 300 (67.7%) |
| | Wireless internet access | 896 (62.8%) | 596 (62.5%) | 85 (63.0%) | 543 (60.5%) | 138 (71.5%) | 226 (51.0%) |
| | None | 29 ( 2.0%) | 20 ( 2.1%) | 3 ( 2.2%) | 20 ( 2.2%) | 3 ( 1.6%) | 13 ( 2.9%) |
| Uses electronic patient records to manage clinical/patient care data (as opposed to billing/scheduling) | | | | | | | |
| | Yes | 825 (58.2%) | 494 (51.8%) | 97 (72.9%) | 477 (53.2%) | 116 (60.7%) | 297 (67.7%) |
| | No | 592 (41.8%) | 459 (48.2%) | 36 (27.1%) | 420 (46.8%) | 75 (39.3%) | 142 (32.3%) |
| | Total | 1417 (100.0%) | 953 (100.0%) | 133 (100.0%) | 897 (100.0%) | 191 (100.0%) | 439 (100.0%) |

**Additional file 1. Characteristics of enrolled practitioners, by practice type (as of January 25, 2013) [unabridged version]**

| | | Dentists | | | | | Hygienists |
|---|---|---|---|---|---|---|---|
| | | Private vs. Non-Private | | | General vs. Specialist | | |
| Characteristic | | All | Private Practice | Non-Private | General Practitioner | Specialist | All |
| | Missing/Blank | 10 | 1 | 2 | 0 | 2 | 4 |
| Brand of electronic patient records software | | | | | | | |
| | Dentrix | 265 (32.3%) | 173 (35.2%) | 15 (15.5%) | 173 (36.3%) | 14 (12.3%) | 129 (43.4%) |
| | Soft Dent | 32 ( 3.9%) | 22 ( 4.5%) | 0 ( 0.0%) | 21 ( 4.4%) | 1 ( 0.9%) | 22 ( 7.4%) |
| | Eagle Soft | 150 (18.3%) | 102 (20.7%) | 5 ( 5.2%) | 96 (20.1%) | 11 ( 9.6%) | 65 (21.9%) |
| | Eagle Dental | 3 ( 0.4%) | 2 ( 0.4%) | 0 ( 0.0%) | 1 ( 0.2%) | 1 ( 0.9%) | 1 ( 0.3%) |
| | Practice Works | 44 ( 5.4%) | 35 ( 7.1%) | 0 ( 0.0%) | 30 ( 6.3%) | 5 ( 4.4%) | 11 ( 3.7%) |
| | GSD Works | 25 ( 3.0%) | 18 ( 3.7%) | 0 ( 0.0%) | 16 ( 3.4%) | 2 ( 1.8%) | 5 ( 1.7%) |
| | Axium | 86 (10.5%) | 21 ( 4.3%) | 44 (45.4%) | 40 ( 8.4%) | 25 (21.9%) | 17 ( 5.7%) |
| | Other | 216 (26.3%) | 119 (24.2%) | 33 (34.0%) | 100 (21.0%) | 55 (48.2%) | 47 (15.8%) |
| | Total | 821 (100.0%) | 492 (100.0%) | 97 (100.0%) | 477 (100.0%) | 114 (100.0%) | 297 (100.0%) |
| | Missing/Blank/NA | 606 | 462 | 38 | 420 | 79 | 146 |
| Of non-EDR users in the next 2 years how likely are you to begin using EDR to manage clinical patient data? | | | | | | | |
| | Very likely | 234 (39.5%) | 178 (38.8%) | 19 (52.8%) | 173 (41.2%) | 24 (32.0%) | 43 (30.5%) |
| | Somewhat likely | 178 (30.1%) | 137 (29.8%) | 10 (27.8%) | 115 (27.4%) | 32 (42.7%) | 38 (27.0%) |
| | Not likely | 110 (18.6%) | 90 (19.6%) | 3 ( 8.3%) | 79 (18.8%) | 14 (18.7%) | 32 (22.7%) |
| | Not sure | 70 (11.8%) | 54 (11.8%) | 4 (11.1%) | 53 (12.6%) | 5 ( 6.7%) | 28 (19.9%) |
| | Total | 592 (100.0%) | 459 (100.0%) | 36 (100.0%) | 420 (100.0%) | 75 (100.0%) | 141 (100.0%) |
| | Missing/Blank/NA | 835 | 495 | 99 | 477 | 118 | 302 |
| Desired level of network participation | | | | | | | |
| | Informational | 114 ( 8.0%) | 71 ( 7.5%) | 13 ( 9.8%) | 67 ( 7.5%) | 16 ( 8.3%) | 29 ( 6.6%) |
| | Limited participation | 304 (21.5%) | 177 (18.7%) | 23 (17.3%) | 166 (18.6%) | 35 (18.2%) | 144 (32.6%) |

**Additional file 1. Characteristics of enrolled practitioners, by practice type (as of January 25, 2013) [unabridged version]**

| | | Dentists | | | | | Hygienists |
|---|---|---|---|---|---|---|---|
| | | Private vs. Non-Private | | | General vs. Specialist | | |
| Characteristic | | All | Private Practice | Non-Private | General Practitioner | Specialist | All |
| | Full participation | 999 (70.5%) | 701 (73.9%) | 97 (72.9%) | 659 (73.9%) | 141 (73.4%) | 269 (60.9%) |
| | Total | 1417 (100.0%) | 949 (100.0%) | 133 (100.0%) | 892 (100.0%) | 192 (100.0%) | 442 (100.0%) |
| | Missing/Blank | 10 | 5 | 2 | 5 | 1 | 1 |
| Type of main practice | | | | | | | |
| | Owner of private practice | 916 (64.6%) | 714 (74.8%) | 0 ( 0.0%) | 595 (66.7%) | 118 (61.1%) | |
| | Associate or employee of a private practice | 176 (12.4%) | 134 (14.0%) | 0 ( 0.0%) | 111 (12.4%) | 23 (11.9%) | |
| | HealthPartners Dental Group | 56 ( 4.0%) | 44 ( 4.6%) | 0 ( 0.0%) | 38 ( 4.3%) | 5 ( 2.6%) | |
| | Permanente Dental Associates | 53 ( 3.7%) | 48 ( 5.0%) | 0 ( 0.0%) | 44 ( 4.9%) | 3 ( 1.6%) | |
| | Other managed care or preferred provider organization | 16 ( 1.1%) | 14 ( 1.5%) | 0 ( 0.0%) | 13 ( 1.5%) | 1 ( 0.5%) | |
| | Public health practice, community health center, or publicly-funded clinic (but not a federal facility) | 58 ( 4.1%) | 0 ( 0.0%) | 38 (28.1%) | 38 ( 4.3%) | 0 ( 0.0%) | |
| | Federal government facility (VA, DoD, Public Health Service) | 24 ( 1.7%) | 0 ( 0.0%) | 18 (13.3%) | 16 ( 1.8%) | 2 ( 1.0%) | |
| | Dental school, academic institution, or facility staffed by dental school | 118 ( 8.3%) | 0 ( 0.0%) | 79 (58.5%) | 37 ( 4.1%) | 41 (21.2%) | |
| | Total | 1417 (100.0%) | 954 (100.0%) | 135 (100.0%) | 892 (100.0%) | 193 (100.0%) | |
| | Missing/Blank | 10 | 0 | 0 | 5 | 0 | |
| Year of graduation from dental school | | | | | | | |
| | Graduation year Mean (S.D.) | 1989.4 (12.0) | 1989.2 (11.7) | 1990.9 (12.1) | 1989.9 (11.9) | 1987.1 (10.5) | |

**Additional file 1. Characteristics of enrolled practitioners, by practice type (as of January 25, 2013) [unabridged version]**

| | | Dentists | | | | | Hygienists |
|---|---|---|---|---|---|---|---|
| | | Private vs. Non-Private | | General vs. Specialist | | | |
| Characteristic | | All | Private Practice | Non-Private | General Practitioner | Specialist | All |
| Country of dental school graduates | | | | | | | |
| | United States | 1349 (94.9%) | 928 (97.5%) | 103 (76.3%) | 865 (96.4%) | 169 (88.0%) | |
| | Canada | 9 ( 0.6%) | 6 ( 0.6%) | 1 ( 0.7%) | 3 ( 0.3%) | 4 ( 2.1%) | |
| | Other | 63 ( 4.4%) | 18 ( 1.9%) | 31 (23.0%) | 29 ( 3.2%) | 19 ( 9.9%) | |
| | Total | 1421 (100.0%) | 952 (100.0%) | 135 (100.0%) | 897 (100.0%) | 192 (100.0%) | |
| | Missing/Blank | 6 | 2 | 0 | 0 | 1 | |
| Type of dentist | | | | | | | |
| | General Dentist | 1148 (80.9%) | 801 (84.2%) | 91 (67.9%) | 897 ( 100%) | 0 ( 0.0%) | |
| | Specialist | 271 (19.1%) | 150 (15.8%) | 43 (32.1%) | 0 ( 0.0%) | 193 ( 100%) | |
| | | 1419 (100.0%) | 951 (100.0%) | 134 (100.0%) | 897 (100.0%) | 193 (100.0%) | |
| | Missing/Blank | 8 | 3 | 1 | 0 | 0 | |
| Among general dentists | | | | | | | |
| | No formal advanced training after dental school | | | | 387 (43.1%) | | |
| | Completed an AEGD program | | | | 94 (10.5%) | | |
| | Completed a GPR program | | | | 199 (22.2%) | | |
| | FAGD | | | | 129 (14.4%) | | |
| | MAGD | | | | 67 ( 7.5%) | | |
| | Completed some other type of advanced training | | | | 188 (21.0%) | | |
| Among dental specialists | | | | | | | |
| | Endodontist | | | | | 23 (11.9%) | |
| | Pediatric dentist | | | | | 43 (22.3%) | |
| | Periodontist | | | | | 45 (23.3%) | |

*Program: \\Westat.com\Dfs\NDPBRN\IT\Systems\Programs\GeneralPrograms\EQReceived\NDPBRN_Enrollment_Freqs.sas -- Run Date: 01/25/2013*

**Additional file 1. Characteristics of enrolled practitioners, by practice type (as of January 25, 2013) [unabridged version]**

| | | Dentists | | | | | Hygienists |
|---|---|---|---|---|---|---|---|
| | | Private vs. Non-Private | | | General vs. Specialist | | |
| Characteristic | | All | Private Practice | Non-Private | General Practitioner | Specialist | All |
| | Prosthodontist | | | | | 16 ( 8.3%) | |
| | Oral & maxillofacial surgeon | | | | | 11 ( 5.7%) | |
| | Orthodontist | | | | | 46 (23.8%) | |
| | Other | | | | | 19 ( 9.8%) | |
| Member of | | | | | | | |
| | American Dental Association | 1133 (79.4%) | 776 (81.3%) | 100 (74.1%) | 716 (79.8%) | 163 (84.5%) | |
| | Academy of General Dentistry | 474 (33.2%) | 344 (36.1%) | 24 (17.8%) | 361 (40.2%) | 8 ( 4.1%) | |
| Patient visits per week | | | | | | | |
| | Number of patient visits Mean (S.D.) | 45.7 (34.9) | 47.6 (32.9) | 31.3 (27.4) | 42.3 (21.5) | 61.1 (60.2) | |
| Frequency of performing the following procedures in a typical month | | | | | | | |
| Nonimplant restorative | Not at all | 176 (12.5%) | 97 (10.3%) | 31 (23.5%) | 20 ( 2.2%) | 107 (57.8%) | |
| | Occasionally | 42 ( 3.0%) | 19 ( 2.0%) | 10 ( 7.6%) | 15 ( 1.7%) | 14 ( 7.6%) | |
| | Routinely | 1189 (84.5%) | 828 (87.7%) | 91 (68.9%) | 857 (96.1%) | 64 (34.6%) | |
| | Total | 1407 (100.0%) | 944 (100.0%) | 132 (100.0%) | 892 (100.0%) | 185 (100.0%) | |
| | Missing/Blank | 20 | 10 | 3 | 5 | 8 | |
| | | | | | | | |
| Implants (prosthetic and surgical procedures) | Not at all | 617 (44.1%) | 391 (41.6%) | 80 (61.5%) | 359 (40.5%) | 112 (60.9%) | |
| | Occasionally | 469 (33.5%) | 325 (34.6%) | 26 (20.0%) | 339 (38.3%) | 14 ( 7.6%) | |
| | Routinely | 313 (22.4%) | 223 (23.7%) | 24 (18.5%) | 188 (21.2%) | 58 (31.5%) | |
| | Total | 1399 (100.0%) | 939 (100.0%) | 130 (100.0%) | 886 (100.0%) | 184 (100.0%) | |
| | Missing/Blank | 28 | 15 | 5 | 11 | 9 | |

**Additional file 1. Characteristics of enrolled practitioners, by practice type (as of January 25, 2013) [unabridged version]**

| | | Dentists | | | | | Hygienists |
|---|---|---|---|---|---|---|---|
| | | Private vs. Non-Private | | | General vs. Specialist | | |
| Characteristic | | All | Private Practice | Non-Private | General Practitioner | Specialist | All |
| | | | | | | | |
| Removable prosthetics | Not at all | 270 (19.3%) | 153 (16.3%) | 51 (38.6%) | 58 ( 6.5%) | 145 (80.1%) | |
| | Occasionally | 499 (35.6%) | 351 (37.3%) | 33 (25.0%) | 368 (41.3%) | 18 ( 9.9%) | |
| | Routinely | 633 (45.1%) | 436 (46.4%) | 48 (36.4%) | 466 (52.2%) | 18 ( 9.9%) | |
| | Total | 1402 (100.0%) | 940 (100.0%) | 132 (100.0%) | 892 (100.0%) | 181 (100.0%) | |
| | Missing/Blank | 25 | 14 | 3 | 5 | 12 | |
| | | | | | | | |
| Extractions (surgical and non-surgical) | Not at all | 151 (10.8%) | 76 ( 8.1%) | 29 (22.5%) | 42 ( 4.7%) | 62 (33.7%) | |
| | Occasionally | 394 (28.2%) | 274 (29.1%) | 24 (18.6%) | 263 (29.7%) | 36 (19.6%) | |
| | Routinely | 854 (61.0%) | 590 (62.8%) | 76 (58.9%) | 581 (65.6%) | 86 (46.7%) | |
| | Total | 1399 (100.0%) | 940 (100.0%) | 129 (100.0%) | 886 (100.0%) | 184 (100.0%) | |
| | Missing/Blank | 28 | 14 | 6 | 11 | 9 | |
| | | | | | | | |
| Periodontal therapy (nonsurgical) | Not at all | 642 (45.8%) | 422 (44.9%) | 54 (41.2%) | 370 (41.6%) | 106 (57.9%) | |
| | Occasionally | 464 (33.1%) | 318 (33.8%) | 39 (29.8%) | 326 (36.7%) | 31 (16.9%) | |
| | Routinely | 295 (21.1%) | 200 (21.3%) | 38 (29.0%) | 193 (21.7%) | 46 (25.1%) | |
| | Total | 1401 (100.0%) | 940 (100.0%) | 131 (100.0%) | 889 (100.0%) | 183 (100.0%) | |
| | Missing/Blank | 26 | 14 | 4 | 8 | 10 | |
| | | | | | | | |
| Periodontal therapy (surgical) | Not at all | 957 (68.9%) | 631 (67.4%) | 94 (72.3%) | 606 (68.6%) | 120 (65.6%) | |
| | Occasionally | 321 (23.1%) | 235 (25.1%) | 20 (15.4%) | 238 (26.9%) | 17 ( 9.3%) | |
| | Routinely | 111 ( 8.0%) | 70 ( 7.5%) | 16 (12.3%) | 40 ( 4.5%) | 46 (25.1%) | |
| | Total | 1389 (100.0%) | 936 (100.0%) | 130 (100.0%) | 884 (100.0%) | 183 (100.0%) | |
| | Missing/Blank | 38 | 18 | 5 | 13 | 10 | |

**Additional file 1. Characteristics of enrolled practitioners, by practice type (as of January 25, 2013) [unabridged version]**

| | | Dentists | | | | | Hygienists |
|---|---|---|---|---|---|---|---|
| | | Private vs. Non-Private | | | General vs. Specialist | | |
| Characteristic | | All | Private Practice | Non-Private | General Practitioner | Specialist | All |
| | | | | | | | |
| Endodontic therapy (anteriors/premolars) | Not at all | 371 (26.4%) | 208 (22.1%) | 59 (44.7%) | 132 (14.8%) | 134 (72.4%) | |
| | Occasionally | 322 (22.9%) | 202 (21.4%) | 37 (28.0%) | 219 (24.6%) | 21 (11.4%) | |
| | Routinely | 711 (50.6%) | 533 (56.5%) | 36 (27.3%) | 540 (60.6%) | 30 (16.2%) | |
| | Total | 1404 (100.0%) | 943 (100.0%) | 132 (100.0%) | 891 (100.0%) | 185 (100.0%) | |
| | Missing/Blank | 23 | 11 | 3 | 6 | 8 | |
| | | | | | | | |
| Endodontic therapy (molars) | Not at all | 618 (44.1%) | 365 (38.8%) | 88 (67.2%) | 317 (35.6%) | 136 (74.3%) | |
| | Occasionally | 336 (24.0%) | 247 (26.2%) | 24 (18.3%) | 255 (28.7%) | 18 ( 9.8%) | |
| | Routinely | 446 (31.9%) | 329 (35.0%) | 19 (14.5%) | 318 (35.7%) | 29 (15.8%) | |
| | Total | 1400 (100.0%) | 941 (100.0%) | 131 (100.0%) | 890 (100.0%) | 183 (100.0%) | |
| | Missing/Blank | 27 | 13 | 4 | 7 | 10 | |
| | | | | | | | |
| Procedures for esthetic reasons only | Not at all | 226 (16.1%) | 111 (11.8%) | 47 (35.6%) | 41 ( 4.6%) | 116 (63.7%) | |
| | Occasionally | 473 (33.7%) | 316 (33.5%) | 46 (34.8%) | 334 (37.4%) | 31 (17.0%) | |
| | Routinely | 706 (50.2%) | 516 (54.7%) | 39 (29.5%) | 519 (58.1%) | 35 (19.2%) | |
| | Total | 1405 (100.0%) | 943 (100.0%) | 132 (100.0%) | 894 (100.0%) | 182 (100.0%) | |
| | Missing/Blank | 22 | 11 | 3 | 3 | 11 | |
| | | | | | | | |
| Orthodontic treatment | Not at all | 975 (69.5%) | 629 (66.9%) | 112 (85.5%) | 637 (71.8%) | 103 (55.7%) | |
| | Occasionally | 252 (18.0%) | 185 (19.7%) | 11 ( 8.4%) | 173 (19.5%) | 25 (13.5%) | |
| | Routinely | 175 (12.5%) | 126 (13.4%) | 8 ( 6.1%) | 77 ( 8.7%) | 57 (30.8%) | |
| | Total | 1402 (100.0%) | 940 (100.0%) | 131 (100.0%) | 887 (100.0%) | 185 (100.0%) | |
| | Missing/Blank | 25 | 14 | 4 | 10 | 8 | |

**Additional file 1. Characteristics of enrolled practitioners, by practice type (as of January 25, 2013) [unabridged version]**

| | | Dentists | | | | | Hygienists |
|---|---|---|---|---|---|---|---|
| | | Private vs. Non-Private | | | General vs. Specialist | | |
| Characteristic | | All | Private Practice | Non-Private | General Practitioner | Specialist | All |
| Educational setting for your dental hygiene training | | | | | | | |
| | Technical or community college | | | | | | 203 (46.1%) |
| | Four-year college | | | | | | 179 (40.7%) |
| | Alabama Dental Hygiene Program | | | | | | 25 ( 5.7%) |
| | Other | | | | | | 33 ( 7.5%) |
| | Total | | | | | | 440 (100.0%) |
| | Missing/Blank | | | | | | 3 |
| Year at which became licensed as a dental hygienist | | | | | | | |
| | Year licensed - Mean (S.D.) | | | | | | 1994.7 (12.6) |
| Highest degree obtained | | | | | | | |
| | Certificate | | | | | | 30 ( 6.8%) |
| | Associate | | | | | | 188 (42.6%) |
| | Baccalaureate | | | | | | 160 (36.3%) |
| | Masters | | | | | | 49 (11.1%) |
| | PhD | | | | | | 5 ( 1.1%) |
| | Other | | | | | | 9 ( 2.0%) |
| | Total | | | | | | 441 (100.0%) |
| | Missing/Blank | | | | | | 2 |
| Member of: | | | | | | | |
| | American Dental Hygienists Association | | | | | | 183 (41.3%) |

**Additional file 1. Characteristics of enrolled practitioners, by practice type (as of January 25, 2013) [unabridged version]**

| | | Dentists | | | | | Hygienists |
|---|---|---|---|---|---|---|---|
| | | Private vs. Non-Private | | | General vs. Specialist | | |
| Characteristic | | All | Private Practice | Non-Private | General Practitioner | Specialist | All |
| Percentage of patients you personally perform this procedure - Mean (S.D.) | | | | | | | |
| | Prophylaxis | | | | | | 73.1 (28.6) |
| | Periodontal therapy | | | | | | 38.2 (33.5) |
| | Subgingival antimicrobial placement | | | | | | 17.6 (30.7) |
| | Restorative functions | | | | | | 0.9 ( 7.5) |
| | Local anesthesia (injection) | | | | | | 8.3 (18.2) |
| | Local anesthesia (subgingival with gel) | | | | | | 16.2 (26.5) |
| | Dental sealants | | | | | | 20.3 (29.7) |
| | Dentinal desensitizers | | | | | | 22.2 (30.2) |
| | Radiographs | | | | | | 65.8 (31.2) |
| | Patient education (in-office) | | | | | | 83.1 (28.3) |
| | Tobacco cessation counseling | | | | | | 25.2 (31.5) |
| | Dietary counseling | | | | | | 34.9 (36.1) |
| | Other | | | | | | 26.0 (39.5) |
