# Supplementary material for: Provision of specific dental procedures by general dentists in the National Dental Practice-Based Research Network: questionnaire findings
Source: BMC Oral Health. 2015 Jan 22;15:11. doi: 10.1186/1472-6831-15-11 (PMC4324862; doi:10.1186/1472-6831-15-11)
Supplement: Supplementary file 2 — Additional file 2: Frequency of provision of procedure types, by dentist, practice, and patient characteristics. Bivariate cross-tabulations. (PDF 959 KB) [file 12903_2014_496_MOESM2_ESM.pdf]

Additional file 2. Frequency of provision of procedure types, by dentist, practice, and patient characteristics

Frequency of provision of three of the five 'very common' procedure types, by dentist, practice, and patient characteristics [a,b]

	Non-implant restorative procedures				Esthetic procedures				Extractions			
Characteristic of the dentist, practice, or patient population	None	Occasionally	Routinely	Total	None	Occasionally	Routinely	Total	None	Occasionally	Routinely	Total
Dentist gender												
Male	34 (2.0)	33 (1.9)	1629 (96.0)	1696	65 (3.8)	573 (33.7)	1062 (62.5)	1700	82 (4.8)	498 (29.4)	1112 (65.7)	1692
Female	15 (2.4)	8 (1.3)	593 (96.3)	616	44 (7.1)	258 (41.8)	315 (51.1)	617	36 (5.9)	226 (36.7)	353 (57.4)	615
Missing/Blank	0	0	11	55	0	4	7	50	1	2	8	60
P-value				0.491				<.0001				0.001
Dentist age in years												
Mean (S.D.)	53.9 (13.6)	55.2 (13.8)	51.0 (11.9)		50.1 (14.0)	48.8 (12.6)	52.6 (11.3)		55.1 (12.1)	52.5 (11.6)	50.1 (12.1)	
P-value				0.023				<.0001				<.0001
Dentist Hispanic/Latino ethnicity												
Yes	5 (4.2)	0 (0.0)	113 (95.8)	118	5 (4.3)	48 (41.0)	64 (54.7)	117	3 (2.5)	36 (30.5)	79 (66.9)	118
No	44 (2.0)	40 (1.8)	2094 (96.1)	2178	102 (4.7)	780 (35.7)	1302 (59.6)	2184	115 (5.3)	681 (31.3)	1378 (63.4)	2174
Missing/Blank	0	1	26	71	2	7	18	66	1	9	16	75
P-value				0.099				0.510				0.429
Dentist race												
White/Caucasian	36 (1.9)	30 (1.6)	1868 (96.6)	1934	83 (4.3)	693 (35.7)	1163 (60.0)	1939	108 (5.6)	636 (33.0)	1185 (61.4)	1929
Black/African-American	4 (3.6)	6 (5.4)	101 (91.0)	111	8 (7.1)	34 (30.4)	70 (62.5)	112	2 (1.8)	15 (13.4)	95 (84.8)	112
American Indian/Alaska Native	1 (16.7)	0 (0.0)	5 (83.3)	6	1 (16.7)	4 (66.7)	1 (16.7)	6	2 (33.3)	2 (33.3)	2 (33.3)	6
Asian	4 (2.3)	4 (2.3)	167 (95.4)	175	10 (5.7)	65 (37.1)	100 (57.1)	175	3 (1.7)	45 (25.7)	127 (72.6)	175
Native Hawaiian/Pacific Islander	1 (50.0)	0 (0.0)	1 (50.0)	2	1 (50.0)	0 (0.0)	1 (50.0)	2	1 (50.0)	0 (0.0)	1 (50.0)	2
Other	3 (4.5)	0 (0.0)	64 (95.5)	67	4 (6.1)	25 (37.9)	37 (56.1)	66	2 (3.0)	19 (28.4)	46 (68.7)	67
Missing/Blank	0	1	27	72	2	14	12	67	1	9	17	76
P-value				0.003				0.071				<.0001
Year of graduation from dental school												
Mean (S.D.)	1986 (13.0)	1986 (14.1)	1990 (12.4)		1991 (14.4)	1992 (12.9)	1988 (11.8)		1985 (12.0)	1988 (12.1)	1991 (12.5)	
P-value				0.030				<.0001				<.0001

	Non-implant restorative procedures				Esthetic procedures				Extractions			
Characteristic of the dentist, practice, or patient population	None	Occasionally	Routinely	Total	None	Occasionally	Routinely	Total	None	Occasionally	Routinely	Total
After dental school: No formal training program												
Yes	11 (1.1)	14 (1.4)	990 (97.5)	1015	45 (4.4)	418 (41.1)	555 (54.5)	1018	46 (4.5)	330 (32.6)	635 (62.8)	1011
No	38 (2.9)	27 (2.1)	1243 (95.0)	1308	64 (4.9)	417 (31.8)	829 (63.3)	1310	73 (5.6)	396 (30.3)	838 (64.1)	1307
Missing/Blank	0	0	0	44	0	0	0	39	0	0	0	49
P-value				0.003				<.0001				0.311
After dental school: Completed an AEGD program												
Yes	5 (2.3)	4 (1.8)	213 (95.9)	222	11 (5.0)	83 (37.4)	128 (57.7)	222	11 (5.0)	62 (28.1)	148 (67.0)	221
No	44 (2.1)	37 (1.8)	2020 (96.1)	2101	98 (4.7)	752 (35.7)	1256 (59.6)	2106	108 (5.2)	664 (31.7)	1325 (63.2)	2097
Missing/Blank	0	0	0	44	0	0	0	39	0	0	0	49
P-value				0.919				0.833				0.534
After dental school: Completed a GPR program												
Yes	10 (2.0)	8 (1.6)	494 (96.5)	512	24 (4.7)	185 (35.9)	306 (59.4)	515	20 (3.9)	150 (29.2)	343 (66.9)	513
No	39 (2.2)	33 (1.8)	1739 (96.0)	1811	85 (4.7)	650 (35.9)	1078 (59.5)	1813	99 (5.5)	576 (31.9)	1130 (62.6)	1805
Missing/Blank	0	0	0	44	0	0	0	39	0	0	0	49
P-value				0.944				1.000				0.138
After dental school: I am a FAGD												
Yes	4 (1.2)	7 (2.2)	313 (96.6)	324	8 (2.5)	89 (27.5)	227 (70.1)	324	18 (5.6)	107 (33.0)	199 (61.4)	324
No	45 (2.3)	34 (1.7)	1920 (96.0)	1999	101 (5.0)	746 (37.2)	1157 (57.7)	2004	101 (5.1)	619 (31.0)	1274 (63.9)	1994
Missing/Blank	0	0	0	44	0	0	0	39	0	0	0	49
P-value				0.405				0.001				0.670
After dental school: Completed MAGD												
Yes	2 (1.5)	1 (0.8)	129 (97.7)	132	2 (1.5)	35 (26.5)	95 (72.0)	132	4 (3.0)	46 (34.8)	82 (62.1)	132
No	47 (2.1)	40 (1.8)	2104 (96.0)	2191	107 (4.9)	800 (36.4)	1289 (58.7)	2196	115 (5.3)	680 (31.1)	1391 (63.6)	2186
Missing/Blank	0	0	0	44	0	0	0	39	0	0	0	49
P-value				0.871				0.007				0.459

	Non-implant restorative procedures				Esthetic procedures				Extractions			
Characteristic of the dentist, practice, or patient population	None	Occasionally	Routinely	Total	None	Occasionally	Routinely	Total	None	Occasionally	Routinely	Total
After dental school: Completed some other training program												
Yes	24 (4.7)	12 (2.4)	473 (92.9)	509	35 (6.9)	135 (26.5)	339 (66.6)	509	39 (7.7)	152 (30.0)	316 (62.3)	507
No	25 (1.4)	29 (1.6)	1760 (97.0)	1814	74 (4.1)	700 (38.5)	1045 (57.4)	1819	80 (4.4)	574 (31.7)	1157 (63.9)	1811
Missing/Blank	0	0	0	44	0	0	0	39	0	0	0	49
P-value				<.0001				<.0001				0.018
Member of: American Dental Association												
Yes	30 (1.6)	30 (1.6)	1767 (96.7)	1827	64 (3.5)	641 (35.0)	1126 (61.5)	1831	90 (4.9)	595 (32.6)	1138 (62.4)	1823
No	19 (3.8)	11 (2.2)	466 (94.0)	496	45 (9.1)	194 (39.0)	258 (51.9)	497	29 (5.9)	131 (26.5)	335 (67.7)	495
Missing/Blank	0	0	0	44	0	0	0	39	0	0	0	49
P-value				0.010				<.0001				0.028
Member of: Academy of General Dentistry												
Yes	9 (1.0)	16 (1.8)	855 (97.2)	880	23 (2.6)	291 (33.0)	568 (64.4)	882	40 (4.5)	283 (32.2)	557 (63.3)	880
No	40 (2.8)	25 (1.7)	1378 (95.5)	1443	86 (5.9)	544 (37.6)	816 (56.4)	1446	79 (5.5)	443 (30.8)	916 (63.7)	1438
Missing/Blank	0	0	0	44	0	0	0	39	0	0	0	49
P-value				0.011				<.0001				0.539
Hours in practice in patient contact												
32 or more hours	20 (1.0)	26 (1.3)	1884 (97.6)	1930	65 (3.4)	676 (35.0)	1191 (61.6)	1932	75 (3.9)	567 (29.4)	1284 (66.7)	1926
Less than 32 hours	29 (7.8)	15 (4.0)	330 (88.2)	374	44 (11.7)	151 (40.2)	181 (48.1)	376	44 (11.8)	153 (41.0)	176 (47.2)	373
Missing/Blank	0	0	19	63	0	8	12	59	0	6	13	68
P-value				<.0001				<.0001				<.0001
Number of different locations at which you see patients												
1 location	36 (1.8)	27 (1.4)	1910 (96.8)	1973	72 (3.6)	707 (35.8)	1198 (60.6)	1977	106 (5.4)	652 (33.1)	1214 (61.6)	1972
2 locations	5 (1.8)	9 (3.2)	263 (94.9)	277	23 (8.3)	96 (34.5)	159 (57.2)	278	8 (2.9)	65 (23.6)	202 (73.5)	275
3 locations	2 (4.7)	3 (7.0)	38 (88.4)	43	6 (14.0)	18 (41.9)	19 (44.2)	43	2 (4.9)	3 (7.3)	36 (87.8)	41
More than 3	6 (20.0)	2 (6.7)	22 (73.3)	30	8 (26.7)	14 (46.7)	8 (26.7)	30	3 (10.0)	6 (20.0)	21 (70.0)	30
Missing/Blank	0	0	0	44	0	0	0	39	0	0	0	49
P-value				<.0001				<.0001				<.0001

	Non-implant restorative procedures				Esthetic procedures				Extractions			
Characteristic of the dentist, practice, or patient population	None	Occasionally	Routinely	Total	None	Occasionally	Routinely	Total	None	Occasionally	Routinely	Total
Practice location												
Inner City of Urban Area	13 (4.7)	6 (2.2)	255 (93.1)	274	26 (9.4)	93 (33.7)	157 (56.9)	276	19 (7.0)	64 (23.4)	190 (69.6)	273
Urban (not inner city)	23 (3.6)	10 (1.5)	614 (94.9)	647	37 (5.7)	251 (38.7)	361 (55.6)	649	43 (6.7)	227 (35.2)	375 (58.1)	645
Suburban	9 (0.9)	14 (1.4)	998 (97.7)	1021	26 (2.5)	341 (33.4)	654 (64.1)	1021	46 (4.5)	365 (35.9)	607 (59.6)	1018
Rural	4 (1.1)	10 (2.7)	357 (96.2)	371	20 (5.4)	148 (39.8)	204 (54.8)	372	10 (2.7)	68 (18.3)	294 (79.0)	372
Missing/Blank	0	1	9	54	0	2	8	49	1	2	7	59
P-value				<.0001				<.0001				<.0001
Type of main practice (full)												
Owner of private practice	17 (1.0)	17 (1.0)	1613 (97.9)	1647	24 (1.5)	514 (31.2)	1109 (67.3)	1647	71 (4.3)	561 (34.2)	1009 (61.5)	1641
Associate or employee of a private practice	7 (2.2)	8 (2.5)	302 (95.3)	317	22 (6.9)	128 (40.1)	169 (53.0)	319	18 (5.7)	101 (32.0)	197 (62.3)	316
HealthPartners Dental Group	2 (4.0)	1 (2.0)	47 (94.0)	50	3 (5.9)	32 (62.7)	16 (31.4)	51	0 (0.0)	6 (12.0)	44 (88.0)	50
Permanente Dental Associates	1 (1.6)	1 (1.6)	61 (96.8)	63	4 (6.3)	47 (74.6)	12 (19.0)	63	1 (1.6)	11 (17.5)	51 (81.0)	63
Other managed care or preferred provider organization	0 (0.0)	1 (5.9)	16 (94.1)	17	2 (11.8)	6 (35.3)	9 (52.9)	17	0 (0.0)	5 (29.4)	12 (70.6)	17
Public health practice, community health center, or publicly-funded clinic (but not a federal facili	7 (6.0)	7 (6.0)	102 (87.9)	116	30 (25.4)	56 (47.5)	32 (27.1)	118	3 (2.5)	15 (12.7)	100 (84.7)	118
Federal government facility (VA, DoD, Public Health Service)	0 (0.0)	2 (5.1)	37 (94.9)	39	7 (17.9)	15 (38.5)	17 (43.6)	39	2 (5.1)	11 (28.2)	26 (66.7)	39
Dental school, academic institution, or facility staffed by dental school	15 (22.4)	3 (4.5)	49 (73.1)	67	17 (25.4)	33 (49.3)	17 (25.4)	67	22 (32.8)	14 (20.9)	31 (46.3)	67
Missing/Blank	0	1	6	51	0	4	3	46	2	2	3	56
P-value				<.0001				<.0001				<.0001
Type of main practice (private)												
Private	27 (1.3)	28 (1.3)	2039 (97.4)	2094	55 (2.6)	727 (34.7)	1315 (62.7)	2097	90 (4.3)	684 (32.8)	1313 (62.9)	2087
Non private	22 (9.9)	12 (5.4)	188 (84.7)	222	54 (24.1)	104 (46.4)	66 (29.5)	224	27 (12.1)	40 (17.9)	157 (70.1)	224
Missing/Blank	0	1	6	51	0	4	3	46	2	2	3	56
P-value				<.0001				<.0001				<.0001
At the same location: Not applicable-only GDs at this location												
Yes	23 (1.2)	30 (1.6)	1879 (97.3)	1932	65 (3.4)	687 (35.5)	1182 (61.1)	1934	77 (4.0)	619 (32.1)	1231 (63.9)	1927

	Non-implant restorative procedures				Esthetic procedures				Extractions			
Characteristic of the dentist, practice, or patient population	None	Occasionally	Routinely	Total	None	Occasionally	Routinely	Total	None	Occasionally	Routinely	Total
No	26 (6.6)	11 (2.8)	354 (90.5)	391	44 (11.2)	148 (37.6)	202 (51.3)	394	42 (10.7)	107 (27.4)	242 (61.9)	391
Missing/Blank	0	0	0	44	0	0	0	39	0	0	0	49
P-value				<.0001				<.0001				<.0001
At the same location: Endodontist												
Yes	6 (5.5)	5 (4.6)	98 (89.9)	109	7 (6.3)	50 (45.0)	54 (48.6)	111	13 (11.7)	33 (29.7)	65 (58.6)	111
No	43 (1.9)	36 (1.6)	2135 (96.4)	2214	102 (4.6)	785 (35.4)	1330 (60.0)	2217	106 (4.8)	693 (31.4)	1408 (63.8)	2207
Missing/Blank	0	0	0	44	0	0	0	39	0	0	0	49
P-value				0.003				0.053				0.014
At the same location: Oral & Maxillofacial Surgeon												
Yes	7 (7.8)	6 (6.7)	77 (85.6)	90	11 (12.1)	36 (39.6)	44 (48.4)	91	14 (15.4)	27 (29.7)	50 (54.9)	91
No	42 (1.9)	35 (1.6)	2156 (96.6)	2233	98 (4.4)	799 (35.7)	1340 (59.9)	2237	105 (4.7)	699 (31.4)	1423 (63.9)	2227
Missing/Blank	0	0	0	44	0	0	0	39	0	0	0	49
P-value				<.0001				0.004				0.0004
At the same location: Orthodontist												
Yes	8 (6.6)	5 (4.1)	109 (89.3)	122	10 (8.1)	51 (41.1)	63 (50.8)	124	10 (8.1)	38 (30.6)	76 (61.3)	124
No	41 (1.9)	36 (1.6)	2124 (96.5)	2201	99 (4.5)	784 (35.6)	1321 (59.9)	2204	109 (5.0)	688 (31.4)	1397 (63.7)	2194
Missing/Blank	0	0	0	44	0	0	0	39	0	0	0	49
P-value				0.001				0.055				0.307
At the same location: Pediatric dentist												
Yes	5 (5.0)	3 (3.0)	92 (92.0)	100	14 (13.9)	45 (44.6)	42 (41.6)	101	10 (10.0)	32 (32.0)	58 (58.0)	100
No	44 (2.0)	38 (1.7)	2141 (96.3)	2223	95 (4.3)	790 (35.5)	1342 (60.3)	2227	109 (4.9)	694 (31.3)	1415 (63.8)	2218
Missing/Blank	0	0	0	44	0	0	0	39	0	0	0	49
P-value				0.052				<.0001				0.081
At the same location: Periodontist												
Yes	5 (3.5)	6 (4.3)	130 (92.2)	141	11 (7.7)	58 (40.8)	73 (51.4)	142	13 (9.1)	51 (35.7)	79 (55.2)	143
No	44 (2.0)	35 (1.6)	2103 (96.4)	2182	98 (4.5)	777 (35.5)	1311 (60.0)	2186	106 (4.9)	675 (31.0)	1394 (64.1)	2175
Missing/Blank	0	0	0	44	0	0	0	39	0	0	0	49

	Non-implant restorative procedures				Esthetic procedures				Extractions			
Characteristic of the dentist, practice, or patient population	None	Occasionally	Routinely	Total	None	Occasionally	Routinely	Total	None	Occasionally	Routinely	Total
P-value				0.023				0.062				0.029
At the same location: Prosthodontist												
Yes	6 (8.6)	3 (4.3)	61 (87.1)	70	8 (11.4)	32 (45.7)	30 (42.9)	70	11 (15.7)	21 (30.0)	38 (54.3)	70
No	43 (1.9)	38 (1.7)	2172 (96.4)	2253	101 (4.5)	803 (35.6)	1354 (60.0)	2258	108 (4.8)	705 (31.4)	1435 (63.8)	2248
Missing/Blank	0	0	0	44	0	0	0	39	0	0	0	49
P-value				0.001				0.003				0.002
At the same location: Other												
Yes	13 (24.1)	2 (3.7)	39 (72.2)	54	18 (33.3)	12 (22.2)	24 (44.4)	54	14 (27.5)	6 (11.8)	31 (60.8)	51
No	36 (1.6)	39 (1.7)	2194 (96.7)	2269	91 (4.0)	823 (36.2)	1360 (59.8)	2274	105 (4.6)	720 (31.8)	1442 (63.6)	2267
Missing/Blank	0	0	0	44	0	0	0	39	0	0	0	49
P-value				<.0001				<.0001				<.0001
How long a patient has to wait: for a new patient exam appt in days												
Mean (S.D.)	10.8 (13.3)	12.7 (29.1)	7.1 (11.5)		12.4 (16.7)	8.3 (11.9)	6.3 (11.6)		8.0 (9.3)	6.5 (10.9)	7.6 (12.8)	
P-value				0.002				<.0001				0.107
How long a patient has to wait: for a treatment procedure appt in days												
Mean (S.D.)	11.0 (14.1)	8.9 (10.6)	7.0 (11.6)		10.4 (12.1)	8.3 (10.3)	6.2 (12.4)		7.9 (8.5)	6.2 (8.7)	7.6 (13.2)	
P-value				0.071				<.0001				0.018
How long a patient has to wait: in the waiting room in mins												
Mean (S.D.)	16.8 (19.0)	11.1 (8.7)	8.2 (7.5)		14.6 (15.3)	8.4 (7.1)	8.0 (7.5)		9.8 (13.4)	6.7 (5.3)	9.2 (8.4)	
P-value				<.0001				<.0001				<.0001
Percentage of patients who are: 1-18 years old												
Mean (S.D.)	22.0 (27.4)	15.7 (21.6)	17.6 (15.1)		25.7 (31.3)	19.1 (17.5)	16.3 (11.8)		15.5 (17.9)	16.7 (16.2)	18.3 (14.9)	
P-value				0.125				<.0001				0.018

Characteristic of the dentist, practice, or patient population	Non-implant restorative procedures				Esthetic procedures				Extractions			
	None	Occasionally	Routinely	Total	None	Occasionally	Routinely	Total	None	Occasionally	Routinely	Total
Percentage of patients who are: 19-44 years old												
Mean (S.D.)	31.9 (19.6)	26.9 (17.5)	28.9 (12.2)		27.8 (18.2)	28.3 (12.3)	29.4 (12.0)		29.4 (13.8)	28.4 (12.7)	29.1 (12.2)	
P-value				0.151				0.093				0.396
Percentage of patients who are: 45-64 years old												
Mean (S.D.)	29.7 (17.0)	34.0 (17.9)	33.4 (11.9)		28.9 (17.6)	32.6 (12.1)	34.1 (11.5)		34.0 (13.2)	34.8 (12.6)	32.6 (11.7)	
P-value				0.114				<.0001				0.0003
Percentage of patients who are: 65 or older												
Mean (S.D.)	16.4 (16.8)	23.4 (19.4)	20.1 (11.3)		17.5 (19.1)	20.1 (11.9)	20.2 (10.6)		21.1 (13.8)	20.1 (11.1)	19.9 (11.5)	
P-value				0.020				0.066				0.569
Percentage of patients who are of Hispanic/Latino ethnicity												
Mean (S.D.)	29.5 (28.7)	16.2 (18.6)	15.0 (18.9)		26.0 (26.2)	16.2 (20.3)	14.0 (17.7)		15.1 (19.1)	13.2 (17.1)	16.3 (20.1)	
P-value				<.0001				<.0001				0.003
Percentage of patients whose race is: White/Caucasian												
Mean (S.D.)	62.0 (23.2)	66.0 (24.7)	68.9 (23.3)		57.6 (28.2)	67.9 (24.7)	69.9 (21.9)		74.1 (19.0)	73.2 (20.6)	66.1 (24.4)	
P-value				0.114				<.0001				<.0001
Percentage of patients whose race is: Black/African-American												
Mean (S.D.)	19.1 (16.5)	20.9 (21.5)	16.7 (17.0)		21.5 (21.5)	16.1 (17.3)	16.9 (16.5)		13.0 (12.6)	14.2 (14.3)	18.3 (18.2)	
P-value				0.198				0.011				<.0001
Percentage of patients whose race is: American Indian/Alaska Native												
Mean (S.D.)	2.3 (4.4)	1.0 (1.7)	1.7 (7.1)		3.8 (14.3)	1.7 (7.2)	1.5 (6.0)		1.1 (2.5)	1.2 (5.5)	1.9 (7.9)	
P-value				0.706				0.006				0.080

	Non-implant restorative procedures				Esthetic procedures				Extractions			
Characteristic of the dentist, practice, or patient population	None	Occasionally	Routinely	Total	None	Occasionally	Routinely	Total	None	Occasionally	Routinely	Total
Percentage of patients whose race is: Asian												
Mean (S.D.)	6.3 (6.0)	6.2 (7.6)	6.3 (7.6)		5.3 (6.0)	6.5 (7.8)	6.3 (7.6)		6.1 (5.9)	6.6 (7.0)	6.2 (8.0)	
P-value				0.993				0.324				0.505
Percentage of patients whose race is: Native Hawaiian/Pacific Islander												
Mean (S.D.)	1.0 (2.7)	0.7 (1.9)	0.8 (2.3)		0.6 (1.7)	0.8 (2.6)	0.8 (2.2)		1.1 (2.8)	0.7 (2.1)	0.8 (2.4)	
P-value				0.796				0.672				0.284
Percentage of patients whose race is: Other												
Mean (S.D.)	9.3 (21.5)	5.2 (15.2)	5.7 (14.7)		11.3 (23.8)	6.9 (16.8)	4.6 (12.6)		4.6 (13.8)	4.1 (11.7)	6.7 (16.3)	
P-value				0.263				<.0001				0.001
Percentage of patients: Covered by a private insurance program												
Mean (S.D.)	37.2 (30.6)	40.4 (31.1)	59.6 (24.6)		29.0 (32.2)	58.2 (27.4)	61.4 (21.4)		59.5 (25.0)	62.7 (22.6)	56.9 (26.1)	
P-value				<.0001				<.0001				<.0001
Percentage of patients: Covered by a public program												
Mean (S.D.)	25.6 (29.3)	31.3 (33.7)	13.6 (23.2)		38.8 (37.7)	17.4 (26.2)	10.5 (19.0)		10.8 (20.8)	10.0 (21.7)	16.5 (24.6)	
P-value				<.0001				<.0001				<.0001
Percentage of patients: Not covered by any third party and pays out of pocket												
Mean (S.D.)	30.0 (32.4)	21.7 (22.4)	23.0 (17.0)		19.2 (27.1)	20.4 (16.8)	25.0 (16.8)		26.5 (21.1)	24.5 (16.9)	22.1 (17.4)	
P-value				0.027				<.0001				0.001
Percent of patients: Receiving free care or substantially reduced fees courtesy of this practice												
Mean (S.D.)	5.0 (17.9)	6.7 (13.1)	3.7 (10.2)		12.1 (23.7)	4.0 (10.9)	3.1 (8.0)		3.2 (11.5)	2.7 (7.0)	4.4 (11.7)	
P-value				0.150				<.0001				0.002

	Non-implant restorative procedures				Esthetic procedures				Extractions			
Characteristic of the dentist, practice, or patient population	None	Occasionally	Routinely	Total	None	Occasionally	Routinely	Total	None	Occasionally	Routinely	Total
Percent of patients who come: For one visit only												
Mean (S.D.)	28.4 (29.7)	13.6 (11.9)	8.1 (8.6)		20.8 (24.1)	8.5 (9.1)	7.8 (7.8)		11.5 (17.2)	6.6 (7.0)	9.3 (10.1)	
P-value				<.0001				<.0001				<.0001
Percent of patients who come: Occasionally only when they have an emergency or specific problem												
Mean (S.D.)	15.2 (14.5)	18.5 (13.3)	12.9 (9.3)		17.3 (15.1)	13.8 (9.9)	12.3 (8.7)		11.2 (9.3)	10.4 (7.4)	14.6 (10.5)	
P-value				0.0004				<.0001				<.0001
Percent of patients who come: Irregularly whether or not they have a problem												
Mean (S.D.)	16.2 (16.7)	17.8 (19.8)	16.1 (10.1)		18.2 (16.4)	16.9 (10.6)	15.6 (9.7)		14.8 (12.4)	15.1 (10.0)	16.8 (10.5)	
P-value				0.610				0.003				0.001
Percent of patients who come: Regularly as recommended or whether or not they have a problem												
Mean (S.D.)	40.3 (30.9)	50.1 (27.1)	62.9 (18.9)		43.8 (27.8)	60.8 (19.9)	64.3 (17.9)		62.4 (23.8)	67.8 (17.0)	59.3 (19.9)	
P-value				<.0001				<.0001				<.0001

[a] This table is limited to the 2,367 GDs who reported their generalist/specialist status. This includes enrollments as of October 31, 2013.

[b] P-values for the association between the characteristic and the procedure computed using Fisher's exact test (for categorical variables) and analysis of variance (for numerical variables).

GD: general dentist
AEGD: Advanced Education in General Dentistry
GPR: General Practice Residency
FAGD: Fellow of the Academy of General Dentistry
MAGD: Master of the Academy of General Dentistry

Frequency of provision of two of the five 'very common' procedure types, by dentist, practice, and patient characteristics [a,b]

	Removable prosthetics					Endodontic therapy – anteriors/premolars			
Characteristic of the dentist, practice, or patient population	None	Occasionally	Routinely	Total		None	Occasionally	Routinely	Total
Dentist gender									
Male	82 (4.8)	591 (34.9)	1022 (60.3)	1695		237 (14.0)	392 (23.2)	1063 (62.8)	1692
Female	48 (7.8)	277 (45.0)	291 (47.2)	616		138 (22.4)	210 (34.0)	269 (43.6)	617
Missing/Blank	0	8	3	56		1	3	7	58
P-value				<.0001					<.0001
Dentist age in years									
Mean (S.D.)	48.9 (13.9)	48.8 (11.9)	52.9 (11.7)			53.7 (12.3)	49.4 (12.6)	51.1 (11.6)	
P-value				<.0001					<.0001
Dentist Hispanic/Latino ethnicity									
Yes	8 (6.8)	36 (30.5)	74 (62.7)	118		13 (11.1)	33 (28.2)	71 (60.7)	117
No	121 (5.6)	826 (37.9)	1230 (56.5)	2177		359 (16.5)	563 (25.9)	1254 (57.6)	2176
Missing/Blank	1	14	12	72		4	9	14	74
P-value				0.231					0.306
Dentist race									
White/Caucasian	108 (5.6)	726 (37.5)	1101 (56.9)	1935		329 (17.0)	496 (25.7)	1106 (57.3)	1931
Black/African-American	5 (4.5)	27 (24.1)	80 (71.4)	112		16 (14.3)	31 (27.7)	65 (58.0)	112
American Indian/Alaska Native	1 (16.7)	3 (50.0)	2 (33.3)	6		1 (16.7)	3 (50.0)	2 (33.3)	6
Asian	10 (5.8)	77 (44.5)	86 (49.7)	173		15 (8.6)	45 (25.9)	114 (65.5)	174
Native Hawaiian/Pacific Islander	1 (50.0)	0 (0.0)	1 (50.0)	2		1 (50.0)	0 (0.0)	1 (50.0)	2
Other	3 (4.5)	25 (37.9)	38 (57.6)	66		10 (14.9)	20 (29.9)	37 (55.2)	67
Missing/Blank	2	18	8	73		4	10	14	75
P-value				0.010					0.101
Year of graduation from dental school									
Mean (S.D.)	1991 (13.7)	1992 (12.3)	1988 (12.3)			1986 (12.6)	1991 (13.0)	1989 (12.1)	
P-value				<.0001					<.0001

	Removable prosthetics				Endodontic therapy – anteriors/premolars			
Characteristic of the dentist, practice, or patient population	None	Occasionally	Routinely	Total	None	Occasionally	Routinely	Total
After dental school: No formal training program								
Yes	53 (5.2)	394 (38.8)	568 (56.0)	1015	174 (17.1)	281 (27.7)	561 (55.2)	1016
No	77 (5.9)	482 (36.9)	748 (57.2)	1307	202 (15.5)	324 (24.8)	778 (59.7)	1304
Missing/Blank	0	0	0	45	0	0	0	47
P-value				0.558				0.098
After dental school: Completed an AEGD program								
Yes	14 (6.3)	82 (36.9)	126 (56.8)	222	36 (16.2)	64 (28.8)	122 (55.0)	222
No	116 (5.5)	794 (37.8)	1190 (56.7)	2100	340 (16.2)	541 (25.8)	1217 (58.0)	2098
Missing/Blank	0	0	0	45	0	0	0	47
P-value				0.843				0.576
After dental school: Completed a GPR program								
Yes	25 (4.9)	203 (39.4)	287 (55.7)	515	71 (13.9)	131 (25.6)	310 (60.5)	512
No	105 (5.8)	673 (37.2)	1029 (56.9)	1807	305 (16.9)	474 (26.2)	1029 (56.9)	1808
Missing/Blank	0	0	0	45	0	0	0	47
P-value				0.549				0.202
After dental school: I am a FAGD								
Yes	11 (3.4)	134 (41.5)	178 (55.1)	323	46 (14.2)	69 (21.4)	208 (64.4)	323
No	119 (6.0)	742 (37.1)	1138 (56.9)	1999	330 (16.5)	536 (26.8)	1131 (56.6)	1997
Missing/Blank	0	0	0	45	0	0	0	47
P-value				0.087				0.032
After dental school: Completed MAGD								
Yes	5 (3.8)	45 (34.4)	81 (61.8)	131	13 (9.9)	29 (22.1)	89 (67.9)	131
No	125 (5.7)	831 (37.9)	1235 (56.4)	2191	363 (16.6)	576 (26.3)	1250 (57.1)	2189
Missing/Blank	0	0	0	45	0	0	0	47
P-value				0.440				0.036

	Removable prosthetics				Endodontic therapy – anteriors/premolars			
Characteristic of the dentist, practice, or patient population	None	Occasionally	Routinely	Total	None	Occasionally	Routinely	Total
After dental school: Completed some other training program								
Yes	46 (9.1)	160 (31.6)	301 (59.4)	507	96 (18.9)	109 (21.5)	303 (59.6)	508
No	84 (4.6)	716 (39.4)	1015 (55.9)	1815	280 (15.5)	496 (27.4)	1036 (57.2)	1812
Missing/Blank	0	0	0	45	0	0	0	47
P-value				<.0001				0.013
Member of: American Dental Association								
Yes	87 (4.8)	691 (37.8)	1048 (57.4)	1826	296 (16.2)	466 (25.5)	1063 (58.2)	1825
No	43 (8.7)	185 (37.3)	268 (54.0)	496	80 (16.2)	139 (28.1)	276 (55.8)	495
Missing/Blank	0	0	0	45	0	0	0	47
P-value				0.005				0.499
Member of: Academy of General Dentistry								
Yes	37 (4.2)	354 (40.2)	490 (55.6)	881	132 (15.0)	225 (25.6)	522 (59.4)	879
No	93 (6.5)	522 (36.2)	826 (57.3)	1441	244 (16.9)	380 (26.4)	817 (56.7)	1441
Missing/Blank	0	0	0	45	0	0	0	47
P-value				0.023				0.362
Hours in practice in patient contact								
32 or more hours	72 (3.7)	709 (36.8)	1147 (59.5)	1928	256 (13.3)	477 (24.8)	1193 (61.9)	1926
Less than 32 hours	54 (14.4)	162 (43.2)	159 (42.4)	375	116 (30.9)	123 (32.8)	136 (36.3)	375
Missing/Blank	4	5	10	64	4	5	10	66
P-value				<.0001				<.0001
Number of different locations at which you see patients								
1 location	100 (5.1)	760 (38.5)	1112 (56.4)	1972	322 (16.4)	515 (26.2)	1132 (57.5)	1969
2 locations	18 (6.5)	95 (34.3)	164 (59.2)	277	38 (13.7)	67 (24.1)	173 (62.2)	278
3 locations	5 (11.6)	9 (20.9)	29 (67.4)	43	6 (14.0)	14 (32.6)	23 (53.5)	43
More than 3	7 (23.3)	12 (40.0)	11 (36.7)	30	10 (33.3)	9 (30.0)	11 (36.7)	30
Missing/Blank	0	0	0	45	0	0	0	47
P-value				0.001				0.094

	Removable prosthetics					Endodontic therapy – anteriors/premolars			
Characteristic of the dentist, practice, or patient population	None	Occasionally	Routinely	Total		None	Occasionally	Routinely	Total
Practice location									
Inner City of Urban Area	25 (9.1)	84 (30.5)	166 (60.4)	275		54 (19.6)	71 (25.8)	150 (54.5)	275
Urban (not inner city)	51 (7.9)	254 (39.3)	342 (52.9)	647		131 (20.3)	177 (27.4)	338 (52.3)	646
Suburban	29 (2.8)	439 (43.1)	551 (54.1)	1019		138 (13.6)	261 (25.7)	618 (60.8)	1017
Rural	25 (6.7)	94 (25.3)	252 (67.9)	371		52 (14.0)	93 (25.0)	227 (61.0)	372
Missing/Blank	0	5	5	55		1	3	6	57
P-value				<.0001					0.002
Type of main practice (full)									
Owner of private practice	36 (2.2)	606 (36.9)	1000 (60.9)	1642		242 (14.7)	384 (23.4)	1015 (61.9)	1641
Associate or employee of a private practice	23 (7.2)	144 (45.3)	151 (47.5)	318		57 (18.0)	100 (31.5)	160 (50.5)	317
HealthPartners Dental Group	3 (5.9)	8 (15.7)	40 (78.4)	51		2 (3.9)	9 (17.6)	40 (78.4)	51
Permanente Dental Associates	4 (6.3)	40 (63.5)	19 (30.2)	63		2 (3.2)	14 (22.2)	47 (74.6)	63
Other managed care or preferred provider organization	2 (11.8)	7 (41.2)	8 (47.1)	17		1 (5.9)	5 (29.4)	11 (64.7)	17
Public health practice, community health center, or publicly-funded clinic (but not a federal facili	32 (27.1)	37 (31.4)	49 (41.5)	118		29 (24.6)	52 (44.1)	37 (31.4)	118
Federal government facility (VA, DoD, Public Health Service)	8 (20.5)	6 (15.4)	25 (64.1)	39		6 (15.4)	20 (51.3)	13 (33.3)	39
Dental school, academic institution, or facility staffed by dental school	22 (32.8)	23 (34.3)	22 (32.8)	67		35 (52.2)	18 (26.9)	14 (20.9)	67
Missing/Blank	0	5	2	52		2	3	2	54
P-value				<.0001					<.0001
Type of main practice (private)									
Private	68 (3.3)	805 (38.5)	1218 (58.2)	2091		304 (14.6)	512 (24.5)	1273 (60.9)	2089
Non private	62 (27.7)	66 (29.5)	96 (42.9)	224		70 (31.3)	90 (40.2)	64 (28.6)	224
Missing/Blank	0	5	2	52		2	3	2	54
P-value				<.0001					<.0001
At the same location: Not applicable-only GDs at this location									
Yes	73 (3.8)	726 (37.6)	1130 (58.6)	1929		284 (14.7)	508 (26.4)	1135 (58.9)	1927

	Removable prosthetics					Endodontic therapy – anteriors/premolars			
Characteristic of the dentist, practice, or patient population	None	Occasionally	Routinely	Total		None	Occasionally	Routinely	Total
No	57 (14.5)	150 (38.2)	186 (47.3)	393		92 (23.4)	97 (24.7)	204 (51.9)	393
Missing/Blank	0	0	0	45		0	0	0	47
P-value				<.0001					<.0001
At the same location: Endodontist									
Yes	13 (11.7)	47 (42.3)	51 (45.9)	111		34 (30.6)	30 (27.0)	47 (42.3)	111
No	117 (5.3)	829 (37.5)	1265 (57.2)	2211		342 (15.5)	575 (26.0)	1292 (58.5)	2209
Missing/Blank	0	0	0	45		0	0	0	47
P-value				0.007					<.0001
At the same location: Oral & Maxillofacial Surgeon									
Yes	15 (16.5)	34 (37.4)	42 (46.2)	91		31 (34.1)	21 (23.1)	39 (42.9)	91
No	115 (5.2)	842 (37.7)	1274 (57.1)	2231		345 (15.5)	584 (26.2)	1300 (58.3)	2229
Missing/Blank	0	0	0	45		0	0	0	47
P-value				<.0001					<.0001
At the same location: Orthodontist									
Yes	14 (11.3)	50 (40.3)	60 (48.4)	124		26 (21.0)	36 (29.0)	62 (50.0)	124
No	116 (5.3)	826 (37.6)	1256 (57.1)	2198		350 (15.9)	569 (25.9)	1277 (58.2)	2196
Missing/Blank	0	0	0	45		0	0	0	47
P-value				0.014					0.156
At the same location: Pediatric dentist									
Yes	21 (20.8)	35 (34.7)	45 (44.6)	101		24 (23.8)	21 (20.8)	56 (55.4)	101
No	109 (4.9)	841 (37.9)	1271 (57.2)	2221		352 (15.9)	584 (26.3)	1283 (57.8)	2219
Missing/Blank	0	0	0	45		0	0	0	47
P-value				<.0001					0.092
At the same location: Periodontist									
Yes	17 (12.0)	55 (38.7)	70 (49.3)	142		31 (21.8)	43 (30.3)	68 (47.9)	142
No	113 (5.2)	821 (37.7)	1246 (57.2)	2180		345 (15.8)	562 (25.8)	1271 (58.4)	2178
Missing/Blank	0	0	0	45		0	0	0	47

	Removable prosthetics					Endodontic therapy – anteriors/premolars			
Characteristic of the dentist, practice, or patient population	None	Occasionally	Routinely	Total		None	Occasionally	Routinely	Total
P-value				0.004					0.037
At the same location: Prosthodontist									
Yes	14 (20.0)	26 (37.1)	30 (42.9)	70		21 (30.0)	22 (31.4)	27 (38.6)	70
No	116 (5.2)	850 (37.7)	1286 (57.1)	2252		355 (15.8)	583 (25.9)	1312 (58.3)	2250
Missing/Blank	0	0	0	45		0	0	0	47
P-value				<.0001					0.001
At the same location: Other									
Yes	17 (31.5)	13 (24.1)	24 (44.4)	54		19 (35.2)	12 (22.2)	23 (42.6)	54
No	113 (5.0)	863 (38.1)	1292 (57.0)	2268		357 (15.8)	593 (26.2)	1316 (58.1)	2266
Missing/Blank	0	0	0	45		0	0	0	47
P-value				<.0001					0.002
How long a patient has to wait: for a new patient exam appt in days									
Mean (S.D.)	10.7 (12.8)	6.9 (9.3)	7.3 (13.5)			7.8 (13.3)	7.8 (10.7)	6.9 (12.3)	
P-value				0.005					0.240
How long a patient has to wait: for a treatment procedure appt in days									
Mean (S.D.)	10.5 (12.2)	6.9 (9.4)	7.0 (13.0)			7.4 (10.4)	7.7 (10.0)	6.9 (12.7)	
P-value				0.006					0.412
How long a patient has to wait: in the waiting room in mins									
Mean (S.D.)	12.8 (10.9)	7.2 (5.8)	8.8 (8.7)			9.6 (11.5)	8.1 (7.2)	8.3 (7.1)	
P-value				<.0001					0.011
Percentage of patients who are: 1-18 years old									
Mean (S.D.)	37.6 (35.5)	18.0 (15.5)	15.6 (10.0)			20.3 (24.0)	17.7 (16.9)	17.0 (11.2)	
P-value				<.0001					0.001

	Removable prosthetics					Endodontic therapy – anteriors/premolars			
Characteristic of the dentist, practice, or patient population	None	Occasionally	Routinely	Total		None	Occasionally	Routinely	Total
Percentage of patients who are: 19-44 years old									
Mean (S.D.)	27.0 (20.5)	29.7 (12.8)	28.5 (11.1)			27.1 (15.0)	29.4 (13.7)	29.2 (11.0)	
P-value				0.018					0.010
Percentage of patients who are: 45-64 years old									
Mean (S.D.)	24.2 (17.7)	33.4 (12.3)	34.2 (11.0)			31.3 (14.6)	33.3 (13.2)	33.9 (10.7)	
P-value				<.0001					0.001
Percentage of patients who are: 65 or older									
Mean (S.D.)	11.2 (10.8)	18.9 (10.4)	21.7 (11.9)			21.3 (15.9)	19.7 (11.9)	19.9 (9.9)	
P-value				<.0001					0.080
Percentage of patients who are of Hispanic/Latino ethnicity									
Mean (S.D.)	28.8 (29.4)	15.5 (18.9)	13.8 (17.7)			16.6 (20.4)	15.8 (19.7)	14.6 (18.6)	
P-value				<.0001					0.173
Percentage of patients whose race is: White/Caucasian									
Mean (S.D.)	56.9 (27.6)	70.2 (22.1)	68.8 (23.3)			67.5 (23.7)	67.1 (24.9)	69.6 (22.4)	
P-value				<.0001					0.064
Percentage of patients whose race is: Black/African-American									
Mean (S.D.)	16.6 (17.8)	14.3 (14.7)	18.5 (18.2)			17.6 (17.2)	17.3 (18.3)	16.4 (16.5)	
P-value				<.0001					0.406
Percentage of patients whose race is: American Indian/Alaska Native									
Mean (S.D.)	3.8 (13.3)	1.7 (7.0)	1.4 (6.1)			1.3 (3.3)	2.0 (8.7)	1.6 (6.9)	
P-value				0.001					0.364

	Removable prosthetics					Endodontic therapy – anteriors/premolars			
Characteristic of the dentist, practice, or patient population	None	Occasionally	Routinely	Total		None	Occasionally	Routinely	Total
Percentage of patients whose race is: Asian									
Mean (S.D.)	7.1 (9.0)	7.2 (7.6)	5.7 (7.3)			6.2 (6.5)	6.3 (7.6)	6.4 (7.9)	
P-value				<.0001					0.868
Percentage of patients whose race is: Native Hawaiian/Pacific Islander									
Mean (S.D.)	1.1 (2.5)	0.9 (2.5)	0.7 (2.1)			0.8 (2.3)	0.7 (1.9)	0.8 (2.5)	
P-value				0.018					0.569
Percentage of patients whose race is: Other									
Mean (S.D.)	14.5 (27.1)	5.7 (14.2)	4.9 (13.3)			6.5 (16.5)	6.7 (16.4)	5.1 (13.7)	
P-value				<.0001					0.057
Percentage of patients: Covered by a private insurance program									
Mean (S.D.)	39.9 (34.5)	63.1 (24.2)	57.8 (23.8)			53.0 (28.4)	56.8 (28.3)	61.3 (22.2)	
P-value				<.0001					<.0001
Percentage of patients: Covered by a public program									
Mean (S.D.)	37.1 (35.9)	11.8 (21.5)	13.6 (22.6)			15.9 (26.9)	17.9 (29.0)	12.1 (19.6)	
P-value				<.0001					<.0001
Percentage of patients: Not covered by any third party and pays out of pocket									
Mean (S.D.)	15.5 (19.9)	21.8 (17.0)	24.6 (17.5)			25.5 (21.4)	21.0 (17.3)	23.3 (16.4)	
P-value				<.0001					0.0004
Percent of patients: Receiving free care or substantially reduced fees courtesy of this practice									
Mean (S.D.)	7.5 (15.9)	3.3 (9.3)	3.9 (10.4)			5.4 (13.9)	4.2 (11.8)	3.3 (8.5)	
P-value				0.0002					0.002

	Removable prosthetics					Endodontic therapy – anteriors/premolars			
Characteristic of the dentist, practice, or patient population	None	Occasionally	Routinely	Total		None	Occasionally	Routinely	Total
Percent of patients who come: For one visit only									
Mean (S.D.)	15.0 (18.9)	7.8 (8.4)	8.6 (9.5)			10.0 (15.4)	8.8 (9.6)	8.1 (7.7)	
P-value				<.0001					0.006
Percent of patients who come: Occasionally only when they have an emergency or specific problem									
Mean (S.D.)	15.5 (13.5)	12.0 (8.7)	13.5 (9.6)			12.8 (11.6)	12.7 (9.5)	13.3 (9.0)	
P-value				<.0001					0.362
Percent of patients who come: Irregularly whether or not they have a problem									
Mean (S.D.)	17.3 (13.9)	15.7 (9.8)	16.3 (10.5)			15.3 (12.2)	16.2 (10.0)	16.4 (10.2)	
P-value				0.192					0.164
Percent of patients who come: Regularly as recommended or whether or not they have a problem									
Mean (S.D.)	52.2 (26.8)	64.5 (18.1)	61.6 (19.5)			61.9 (24.1)	62.4 (19.7)	62.1 (18.2)	
P-value				<.0001					0.947

[a] This table is limited to the 2,367 GDs who reported their generalist/specialist status. This includes enrollments as of October 31, 2013.

[b] P-values for the association between the characteristic and the procedure computed using Fisher's exact test (for categorical variables) and analysis of variance (for numerical variables).

GD:	general dentist
AEGD:	Advanced Education in General Dentistry
GPR:	General Practice Residency
FAGD:	Fellow of the Academy of General Dentistry
MAGD:	Master of the Academy of General Dentistry

Frequency of provision of the three 'common' procedure types, by dentist, practice, and patient characteristics [a,b]

	Endodontic therapy - molars				Implants				Periodontal therapy – non-surgical			
Characteristic of the dentist, practice, or patient population	None	Occasionally	Routinely	Total	None	Occasionally	Routinely	Total	None	Occasionally	Routinely	Total
Dentist gender												
Male	577 (34.0)	427 (25.2)	692 (40.8)	1696	577 (34.3)	674 (40.0)	432 (25.7)	1683	649 (38.3)	649 (38.3)	395 (23.3)	1693
Female	303 (49.7)	172 (28.2)	135 (22.1)	610	307 (50.2)	225 (36.8)	79 (12.9)	611	262 (42.7)	206 (33.6)	145 (23.7)	613
Missing/Blank	3	4	4	61	5	3	3	73	6	4	1	61
P-value				<.0001				<.0001				0.078
Dentist age in years												
Mean (S.D.)	52.1 (12.1)	49.1 (12.5)	51.5 (11.4)		51.5 (13.1)	49.6 (11.6)	53.1 (10.4)		49.7 (12.2)	52.1 (12.0)	52.0 (11.7)	
P-value				<.0001				<.0001				<.0001
Dentist Hispanic/Latino ethnicity												
Yes	43 (37.1)	30 (25.9)	43 (37.1)	116	39 (34.5)	49 (43.4)	25 (22.1)	113	36 (31.3)	46 (40.0)	33 (28.7)	115
No	829 (38.1)	563 (25.9)	782 (36.0)	2174	838 (38.7)	842 (38.9)	485 (22.4)	2165	873 (40.1)	800 (36.8)	502 (23.1)	2175
Missing/Blank	11	10	6	77	12	11	4	89	8	13	6	77
P-value				0.965				0.602				0.134
Dentist race												
White/Caucasian	767 (39.7)	484 (25.1)	681 (35.2)	1932	715 (37.3)	750 (39.1)	453 (23.6)	1918	791 (41.0)	732 (38.0)	405 (21.0)	1928
Black/African-American	38 (34.9)	28 (25.7)	43 (39.4)	109	67 (59.8)	30 (26.8)	15 (13.4)	112	28 (25.0)	43 (38.4)	41 (36.6)	112
American Indian/Alaska Native	5 (83.3)	1 (16.7)	0 (0.0)	6	3 (50.0)	2 (33.3)	1 (16.7)	6	5 (83.3)	1 (16.7)	0 (0.0)	6
Asian	42 (24.3)	56 (32.4)	75 (43.4)	173	66 (38.2)	78 (45.1)	29 (16.8)	173	59 (33.9)	51 (29.3)	64 (36.8)	174
Native Hawaiian/Pacific Islander	1 (50.0)	1 (50.0)	0 (0.0)	2	2 (100)	0 (0.0)	0 (0.0)	2	2 (100)	0 (0.0)	0 (0.0)	2
Other	21 (31.3)	24 (35.8)	22 (32.8)	67	23 (34.3)	30 (44.8)	14 (20.9)	67	20 (29.9)	22 (32.8)	25 (37.3)	67
Missing/Blank	9	9	10	78	13	12	2	89	12	10	6	78
P-value				0.001				<.0001				<.0001
Year of graduation from dental school												
Mean (S.D.)	1988 (12.5)	1992 (12.9)	1989 (11.9)		1989 (13.5)	1991 (12.1)	1987 (11.0)		1991 (12.5)	1988 (12.6)	1989 (12.2)	
P-value				<.0001				<.0001				<.0001

Characteristic of the dentist, practice, or patient population	Endodontic therapy - molars					Implants					Periodontal therapy – non-surgical			
	None	Occasionally	Routinely	Total		None	Occasionally	Routinely	Total		None	Occasionally	Routinely	Total
After dental school: No formal training program														
Yes	423 (41.7)	253 (24.9)	339 (33.4)	1015		457 (45.2)	401 (39.7)	152 (15.0)	1010		437 (43.2)	361 (35.7)	214 (21.1)	1012
No	460 (35.3)	350 (26.9)	492 (37.8)	1302		432 (33.4)	501 (38.7)	362 (28.0)	1295		480 (36.8)	498 (38.2)	327 (25.1)	1305
Missing/Blank	0	0	0	50		0	0	0	62		0	0	0	50
P-value				0.008					<.0001					0.005
After dental school: Completed an AEGD program														
Yes	87 (39.2)	64 (28.8)	71 (32.0)	222		76 (34.2)	94 (42.3)	52 (23.4)	222		90 (40.5)	81 (36.5)	51 (23.0)	222
No	796 (38.0)	539 (25.7)	760 (36.3)	2095		813 (39.0)	808 (38.8)	462 (22.2)	2083		827 (39.5)	778 (37.1)	490 (23.4)	2095
Missing/Blank	0	0	0	50		0	0	0	62		0	0	0	50
P-value				0.387					0.376					0.954
After dental school: Completed a GPR program														
Yes	172 (33.7)	146 (28.6)	192 (37.6)	510		180 (35.5)	200 (39.4)	127 (25.0)	507		181 (35.3)	214 (41.7)	118 (23.0)	513
No	711 (39.3)	457 (25.3)	639 (35.4)	1807		709 (39.4)	702 (39.0)	387 (21.5)	1798		736 (40.8)	645 (35.8)	423 (23.4)	1804
Missing/Blank	0	0	0	50		0	0	0	62		0	0	0	50
P-value				0.059					0.151					0.031
After dental school: I am a FAGD														
Yes	99 (30.7)	71 (22.0)	152 (47.2)	322		87 (26.9)	135 (41.7)	102 (31.5)	324		109 (33.7)	130 (40.2)	84 (26.0)	323
No	784 (39.3)	532 (26.7)	679 (34.0)	1995		802 (40.5)	767 (38.7)	412 (20.8)	1981		808 (40.5)	729 (36.6)	457 (22.9)	1994
Missing/Blank	0	0	0	50		0	0	0	62		0	0	0	50
P-value				<.0001					<.0001					0.069
After dental school: Completed MAGD														
Yes	38 (28.8)	31 (23.5)	63 (47.7)	132		29 (22.0)	55 (41.7)	48 (36.4)	132		48 (36.4)	51 (38.6)	33 (25.0)	132
No	845 (38.7)	572 (26.2)	768 (35.1)	2185		860 (39.6)	847 (39.0)	466 (21.4)	2173		869 (39.8)	808 (37.0)	508 (23.2)	2185
Missing/Blank	0	0	0	50		0	0	0	62		0	0	0	50
P-value				0.013					<.0001					0.721

	Endodontic therapy - molars				Implants				Periodontal therapy – non-surgical			
Characteristic of the dentist, practice, or patient population	None	Occasionally	Routinely	Total	None	Occasionally	Routinely	Total	None	Occasionally	Routinely	Total
After dental school: Completed some other training program												
Yes	186 (36.6)	128 (25.2)	194 (38.2)	508	152 (30.0)	174 (34.4)	180 (35.6)	506	176 (34.6)	183 (36.0)	149 (29.3)	508
No	697 (38.5)	475 (26.3)	637 (35.2)	1809	737 (41.0)	728 (40.5)	334 (18.6)	1799	741 (41.0)	676 (37.4)	392 (21.7)	1809
Missing/Blank	0	0	0	50	0	0	0	62	0	0	0	50
P-value				0.470				<.0001				0.001
Member of: American Dental Association												
Yes	676 (37.1)	485 (26.6)	662 (36.3)	1823	638 (35.2)	734 (40.5)	441 (24.3)	1813	727 (39.9)	676 (37.1)	419 (23.0)	1822
No	207 (41.9)	118 (23.9)	169 (34.2)	494	251 (51.0)	168 (34.1)	73 (14.8)	492	190 (38.4)	183 (37.0)	122 (24.6)	495
Missing/Blank	0	0	0	50	0	0	0	62	0	0	0	50
P-value				0.150				<.0001				0.713
Member of: Academy of General Dentistry												
Yes	312 (35.5)	221 (25.2)	345 (39.3)	878	265 (30.1)	371 (42.2)	244 (27.7)	880	320 (36.4)	357 (40.7)	201 (22.9)	878
No	571 (39.7)	382 (26.5)	486 (33.8)	1439	624 (43.8)	531 (37.3)	270 (18.9)	1425	597 (41.5)	502 (34.9)	340 (23.6)	1439
Missing/Blank	0	0	0	50	0	0	0	62	0	0	0	50
P-value				0.025				<.0001				0.014
Hours in practice in patient contact												
32 or more hours	660 (34.3)	515 (26.8)	749 (38.9)	1924	682 (35.6)	778 (40.6)	455 (23.8)	1915	729 (37.9)	720 (37.4)	476 (24.7)	1925
Less than 32 hours	214 (57.2)	86 (23.0)	74 (19.8)	374	201 (54.2)	116 (31.3)	54 (14.6)	371	182 (48.9)	132 (35.5)	58 (15.6)	372
Missing/Blank	9	2	8	69	6	8	5	81	6	7	7	70
P-value				<.0001				<.0001				<.0001
Number of different locations at which you see patients												
1 location	772 (39.3)	487 (24.8)	707 (36.0)	1966	744 (38.0)	770 (39.4)	442 (22.6)	1956	797 (40.5)	721 (36.6)	450 (22.9)	1968
2 locations	83 (29.9)	91 (32.7)	104 (37.4)	278	103 (37.3)	108 (39.1)	65 (23.6)	276	98 (35.4)	105 (37.9)	74 (26.7)	277
3 locations	14 (32.6)	13 (30.2)	16 (37.2)	43	22 (51.2)	16 (37.2)	5 (11.6)	43	13 (30.2)	17 (39.5)	13 (30.2)	43
More than 3	14 (46.7)	12 (40.0)	4 (13.3)	30	20 (66.7)	8 (26.7)	2 (6.7)	30	9 (31.0)	16 (55.2)	4 (13.8)	29
Missing/Blank	0	0	0	50	0	0	0	62	0	0	0	50
P-value				0.002				0.023				0.162

	Endodontic therapy - molars				Implants				Periodontal therapy – non-surgical			
Characteristic of the dentist, practice, or patient population	None	Occasionally	Routinely	Total	None	Occasionally	Routinely	Total	None	Occasionally	Routinely	Total
Practice location												
Inner City of Urban Area	116 (42.3)	68 (24.8)	90 (32.8)	274	138 (50.5)	92 (33.7)	43 (15.8)	273	96 (34.9)	100 (36.4)	79 (28.7)	275
Urban (not inner city)	285 (44.3)	151 (23.5)	207 (32.2)	643	251 (39.0)	256 (39.8)	136 (21.2)	643	270 (41.7)	229 (35.4)	148 (22.9)	647
Suburban	340 (33.4)	297 (29.1)	382 (37.5)	1019	340 (33.6)	396 (39.2)	275 (27.2)	1011	414 (40.8)	369 (36.4)	231 (22.8)	1014
Rural	140 (37.7)	85 (22.9)	146 (39.4)	371	156 (42.4)	153 (41.6)	59 (16.0)	368	133 (35.8)	159 (42.9)	79 (21.3)	371
Missing/Blank	2	2	6	60	4	5	1	72	4	2	4	60
P-value				0.001				<.0001				0.063
Type of main practice (full)												
Owner of private practice	592 (36.1)	383 (23.4)	665 (40.5)	1640	513 (31.4)	675 (41.3)	445 (27.3)	1633	573 (34.9)	622 (37.9)	445 (27.1)	1640
Associate or employee of a private practice	120 (37.7)	97 (30.5)	101 (31.8)	318	142 (44.9)	132 (41.8)	42 (13.3)	316	159 (50.3)	108 (34.2)	49 (15.5)	316
HealthPartners Dental Group	11 (21.6)	25 (49.0)	15 (29.4)	51	30 (60.0)	17 (34.0)	3 (6.0)	50	38 (74.5)	12 (23.5)	1 (2.0)	51
Permanente Dental Associates	18 (28.6)	31 (49.2)	14 (22.2)	63	36 (57.1)	26 (41.3)	1 (1.6)	63	30 (47.6)	32 (50.8)	1 (1.6)	63
Other managed care or preferred provider organization	4 (23.5)	6 (35.3)	7 (41.2)	17	8 (47.1)	8 (47.1)	1 (5.9)	17	12 (70.6)	4 (23.5)	1 (5.9)	17
Public health practice, community health center, or publicly-funded clinic (but not a federal facili	72 (61.5)	29 (24.8)	16 (13.7)	117	101 (87.1)	14 (12.1)	1 (0.9)	116	49 (41.5)	46 (39.0)	23 (19.5)	118
Federal government facility (VA, DoD, Public Health Service)	16 (42.1)	17 (44.7)	5 (13.2)	38	21 (55.3)	11 (28.9)	6 (15.8)	38	15 (39.5)	15 (39.5)	8 (21.1)	38
Dental school, academic institution, or facility staffed by dental school	47 (71.2)	11 (16.7)	8 (12.1)	66	36 (54.5)	16 (24.2)	14 (21.2)	66	37 (55.2)	18 (26.9)	12 (17.9)	67
Missing/Blank	3	4	0	57	2	3	1	68	4	2	1	57
P-value				<.0001				<.0001				<.0001
Type of main practice (private)												
Private	745 (35.7)	542 (25.9)	802 (38.4)	2089	729 (35.1)	858 (41.3)	492 (23.7)	2079	812 (38.9)	778 (37.3)	497 (23.8)	2087
Non private	135 (61.1)	57 (25.8)	29 (13.1)	221	158 (71.8)	41 (18.6)	21 (9.5)	220	101 (45.3)	79 (35.4)	43 (19.3)	223
Missing/Blank	3	4	0	57	2	3	1	68	4	2	1	57
P-value				<.0001				<.0001				0.137
At the same location: Not applicable-only GDs at this location												
Yes	720 (37.4)	502 (26.1)	704 (36.6)	1926	699 (36.5)	781 (40.7)	437 (22.8)	1917	722 (37.5)	728 (37.8)	476 (24.7)	1926

	Endodontic therapy - molars				Implants				Periodontal therapy – non-surgical			
Characteristic of the dentist, practice, or patient population	None	Occasionally	Routinely	Total	None	Occasionally	Routinely	Total	None	Occasionally	Routinely	Total
No	163 (41.7)	101 (25.8)	127 (32.5)	391	190 (49.0)	121 (31.2)	77 (19.8)	388	195 (49.9)	131 (33.5)	65 (16.6)	391
Missing/Blank	0	0	0	50	0	0	0	62	0	0	0	50
P-value				0.222				<.0001				<.0001
At the same location: Endodontist												
Yes	57 (51.8)	34 (30.9)	19 (17.3)	110	48 (44.0)	37 (33.9)	24 (22.0)	109	61 (55.0)	38 (34.2)	12 (10.8)	111
No	826 (37.4)	569 (25.8)	812 (36.8)	2207	841 (38.3)	865 (39.4)	490 (22.3)	2196	856 (38.8)	821 (37.2)	529 (24.0)	2206
Missing/Blank	0	0	0	50	0	0	0	62	0	0	0	50
P-value				<.0001				0.431				0.0005
At the same location: Oral & Maxillofacial Surgeon												
Yes	44 (48.9)	23 (25.6)	23 (25.6)	90	43 (47.3)	27 (29.7)	21 (23.1)	91	43 (47.3)	35 (38.5)	13 (14.3)	91
No	839 (37.7)	580 (26.0)	808 (36.3)	2227	846 (38.2)	875 (39.5)	493 (22.3)	2214	874 (39.3)	824 (37.0)	528 (23.7)	2226
Missing/Blank	0	0	0	50	0	0	0	62	0	0	0	50
P-value				0.060				0.124				0.079
At the same location: Orthodontist												
Yes	48 (39.0)	34 (27.6)	41 (33.3)	123	60 (49.2)	35 (28.7)	27 (22.1)	122	72 (58.5)	31 (25.2)	20 (16.3)	123
No	835 (38.1)	569 (25.9)	790 (36.0)	2194	829 (38.0)	867 (39.7)	487 (22.3)	2183	845 (38.5)	828 (37.7)	521 (23.7)	2194
Missing/Blank	0	0	0	50	0	0	0	62	0	0	0	50
P-value				0.809				0.023				<.0001
At the same location: Pediatric dentist												
Yes	36 (36.4)	38 (38.4)	25 (25.3)	99	60 (60.6)	22 (22.2)	17 (17.2)	99	56 (56.0)	36 (36.0)	8 (8.0)	100
No	847 (38.2)	565 (25.5)	806 (36.3)	2218	829 (37.6)	880 (39.9)	497 (22.5)	2206	861 (38.8)	823 (37.1)	533 (24.0)	2217
Missing/Blank	0	0	0	50	0	0	0	62	0	0	0	50
P-value				0.010				<.0001				<.0001
At the same location: Periodontist												
Yes	59 (41.8)	47 (33.3)	35 (24.8)	141	55 (39.3)	48 (34.3)	37 (26.4)	140	82 (57.3)	45 (31.5)	16 (11.2)	143
No	824 (37.9)	556 (25.6)	796 (36.6)	2176	834 (38.5)	854 (39.4)	477 (22.0)	2165	835 (38.4)	814 (37.4)	525 (24.1)	2174
Missing/Blank	0	0	0	50	0	0	0	62	0	0	0	50

	Endodontic therapy - molars				Implants				Periodontal therapy – non-surgical			
Characteristic of the dentist, practice, or patient population	None	Occasionally	Routinely	Total	None	Occasionally	Routinely	Total	None	Occasionally	Routinely	Total
P-value				0.012				0.350				<.0001
At the same location: Prosthodontist												
Yes	33 (47.8)	24 (34.8)	12 (17.4)	69	35 (50.7)	20 (29.0)	14 (20.3)	69	34 (48.6)	26 (37.1)	10 (14.3)	70
No	850 (37.8)	579 (25.8)	819 (36.4)	2248	854 (38.2)	882 (39.4)	500 (22.4)	2236	883 (39.3)	833 (37.1)	531 (23.6)	2247
Missing/Blank	0	0	0	50	0	0	0	62	0	0	0	50
P-value				0.003				0.106				0.128
At the same location: Other												
Yes	29 (54.7)	6 (11.3)	18 (34.0)	53	31 (57.4)	9 (16.7)	14 (25.9)	54	26 (49.1)	12 (22.6)	15 (28.3)	53
No	854 (37.7)	597 (26.4)	813 (35.9)	2264	858 (38.1)	893 (39.7)	500 (22.2)	2251	891 (39.4)	847 (37.4)	526 (23.2)	2264
Missing/Blank	0	0	0	50	0	0	0	62	0	0	0	50
P-value				0.014				0.001				0.075
How long a patient has to wait: for a new patient exam appt in days												
Mean (S.D.)	8.5 (15.1)	7.7 (11.0)	5.7 (8.5)		9.0 (16.2)	6.5 (9.0)	5.8 (7.7)		8.0 (14.5)	7.2 (10.0)	6.2 (10.4)	
P-value				<.0001				<.0001				0.024
How long a patient has to wait: for a treatment procedure appt in days												
Mean (S.D.)	8.3 (15.4)	7.6 (9.8)	5.7 (7.8)		8.6 (15.3)	6.6 (9.6)	5.9 (7.4)		8.0 (15.0)	7.2 (9.1)	5.8 (8.6)	
P-value				<.0001				<.0001				0.002
How long a patient has to wait: in the waiting room in mins												
Mean (S.D.)	8.5 (9.0)	8.3 (7.7)	8.4 (7.1)		9.7 (10.0)	7.9 (6.8)	7.0 (5.2)		8.1 (8.2)	8.5 (8.3)	8.9 (7.0)	
P-value				0.832				<.0001				0.243
Percentage of patients who are: 1-18 years old												
Mean (S.D.)	18.3 (19.2)	17.5 (14.7)	17.1 (10.8)		21.3 (21.2)	16.5 (10.7)	13.5 (7.9)		17.3 (14.6)	17.6 (16.2)	18.5 (16.1)	
P-value				0.282				<.0001				0.359

	Endodontic therapy - molars					Implants					Periodontal therapy – non-surgical			
Characteristic of the dentist, practice, or patient population	None	Occasionally	Routinely	Total		None	Occasionally	Routinely	Total		None	Occasionally	Routinely	Total
Percentage of patients who are: 19-44 years old														
Mean (S.D.)	28.0 (13.5)	29.5 (12.5)	29.4 (10.9)			28.1 (13.8)	30.2 (11.5)	28.2 (11.4)			29.5 (12.6)	28.2 (12.1)	28.9 (12.8)	
P-value				0.026					0.001					0.091
Percentage of patients who are: 45-64 years old														
Mean (S.D.)	33.0 (13.5)	33.1 (11.5)	34.0 (10.8)			31.9 (13.8)	33.2 (10.8)	35.9 (10.7)			33.5 (11.7)	33.5 (12.3)	32.7 (12.5)	
P-value				0.193					<.0001					0.426
Percentage of patients who are: 65 or older														
Mean (S.D.)	20.7 (13.4)	20.0 (11.0)	19.5 (9.7)			18.8 (12.5)	20.1 (10.4)	22.4 (11.6)			19.7 (11.4)	20.7 (12.1)	19.8 (11.1)	
P-value				0.098					<.0001					0.167
Percentage of patients who are of Hispanic/Latino ethnicity														
Mean (S.D.)	15.6 (19.9)	14.4 (17.7)	15.3 (19.3)			18.2 (23.0)	13.6 (16.4)	12.8 (15.1)			13.3 (17.2)	14.6 (18.5)	19.2 (22.2)	
P-value				0.507					<.0001					<.0001
Percentage of patients whose race is: White/Caucasian														
Mean (S.D.)	68.6 (23.6)	67.5 (23.7)	69.5 (22.7)			64.6 (26.4)	70.0 (21.4)	73.2 (19.6)			70.8 (21.0)	68.9 (23.7)	64.3 (25.9)	
P-value				0.284					<.0001					<.0001
Percentage of patients whose race is: Black/African-American														
Mean (S.D.)	17.6 (17.9)	16.1 (16.4)	16.5 (16.4)			19.2 (19.8)	16.0 (15.4)	14.2 (13.9)			15.8 (15.3)	16.8 (17.5)	18.7 (18.9)	
P-value				0.185					<.0001					0.007
Percentage of patients whose race is: American Indian/Alaska Native														
Mean (S.D.)	1.3 (4.6)	2.3 (10.0)	1.6 (6.6)			2.1 (9.1)	1.6 (5.7)	1.1 (4.8)			1.4 (4.7)	1.7 (7.1)	2.0 (9.7)	
P-value				0.035					0.038					0.313

	Endodontic therapy - molars				Implants				Periodontal therapy – non-surgical			
Characteristic of the dentist, practice, or patient population	None	Occasionally	Routinely	Total	None	Occasionally	Routinely	Total	None	Occasionally	Routinely	Total
Percentage of patients whose race is: Asian												
Mean (S.D.)	5.8 (6.5)	7.1 (8.2)	6.3 (8.2)		5.9 (8.0)	6.6 (7.5)	6.6 (6.9)		6.4 (6.6)	6.0 (7.0)	6.8 (9.7)	
P-value				0.006				0.157				0.143
Percentage of patients whose race is: Native Hawaiian/Pacific Islander												
Mean (S.D.)	0.7 (2.1)	1.0 (2.4)	0.7 (2.4)		0.7 (2.1)	0.9 (2.6)	0.8 (2.2)		0.7 (2.3)	0.8 (2.3)	0.8 (2.4)	
P-value				0.076				0.288				0.595
Percentage of patients whose race is: Other												
Mean (S.D.)	5.9 (15.2)	6.0 (15.4)	5.4 (14.3)		7.5 (18.4)	5.0 (12.8)	4.2 (10.9)		4.9 (13.2)	5.8 (15.1)	7.4 (17.4)	
P-value				0.728				<.0001				0.010
Percentage of patients: Covered by a private insurance program												
Mean (S.D.)	55.6 (27.2)	60.2 (26.7)	61.5 (20.9)		53.0 (29.4)	63.0 (22.3)	61.6 (19.6)		60.0 (25.4)	59.5 (25.4)	55.8 (24.2)	
P-value				<.0001				<.0001				0.005
Percentage of patients: Covered by a public program												
Mean (S.D.)	15.2 (25.9)	15.8 (25.6)	11.8 (19.1)		21.5 (29.0)	11.4 (19.7)	6.4 (15.4)		14.1 (23.5)	13.3 (23.4)	15.8 (24.8)	
P-value				0.002				<.0001				0.159
Percentage of patients: Not covered by any third party and pays out of pocket												
Mean (S.D.)	24.2 (19.3)	20.7 (17.1)	23.7 (15.9)		19.9 (18.2)	22.6 (16.0)	29.4 (17.5)		22.3 (18.3)	23.1 (17.2)	24.3 (16.9)	
P-value				0.0004				<.0001				0.103
Percent of patients: Receiving free care or substantially reduced fees courtesy of this practice												
Mean (S.D.)	5.1 (13.5)	3.2 (8.7)	3.0 (7.3)		5.6 (14.2)	2.9 (7.6)	2.3 (3.9)		3.5 (10.2)	4.0 (10.4)	4.1 (11.0)	
P-value				<.0001				<.0001				0.481

	Endodontic therapy - molars				Implants				Periodontal therapy – non-surgical			
Characteristic of the dentist, practice, or patient population	None	Occasionally	Routinely	Total	None	Occasionally	Routinely	Total	None	Occasionally	Routinely	Total
Percent of patients who come: For one visit only												
Mean (S.D.)	9.1 (11.8)	8.6 (9.1)	8.1 (7.7)		9.9 (11.7)	8.3 (8.3)	7.0 (9.2)		8.8 (11.6)	8.0 (7.9)	9.3 (9.9)	
P-value				0.130				<.0001				0.053
Percent of patients who come: Occasionally only when they have an emergency or specific problem												
Mean (S.D.)	12.7 (9.9)	13.0 (9.5)	13.4 (9.3)		14.5 (11.4)	12.7 (7.9)	11.3 (8.5)		12.7 (9.5)	13.0 (9.3)	13.8 (10.0)	
P-value				0.338				<.0001				0.119
Percent of patients who come: Irregularly whether or not they have a problem												
Mean (S.D.)	16.2 (11.4)	15.7 (9.4)	16.5 (10.1)		16.4 (11.1)	16.5 (10.0)	15.2 (10.0)		15.9 (10.4)	16.5 (11.0)	16.2 (9.8)	
P-value				0.415				0.059				0.542
Percent of patients who come: Regularly as recommended or whether or not they have a problem												
Mean (S.D.)	62.0 (21.3)	62.7 (19.0)	62.0 (18.2)		59.2 (21.6)	62.5 (18.0)	66.5 (18.1)		62.6 (20.2)	62.6 (19.1)	60.8 (19.6)	
P-value				0.751				<.0001				0.184

[a] This table is limited to the 2,367 GDs who reported their generalist/specialist status. This includes enrollments as of October 31, 2013.

[b] P-values for the association between the characteristic and the procedure computed using Fisher's exact test (for categorical variables) and analysis of variance (for numerical variables).

GD: general dentist
AEGD: Advanced Education in General Dentistry
GPR: General Practice Residency
FAGD: Fellow of the Academy of General Dentistry
MAGD: Master of the Academy of General Dentistry

Frequency of provision of the two 'uncommon' procedure types, by dentist, practice, and patient characteristics [a,b]

	Orthodontic treatment				Surgical periodontal therapy			
Characteristic of the dentist, practice, or patient population	None	Occasionally	Routinely	Total	None	Occasionally	Routinely	Total
Dentist gender								
Male	1122 (66.2)	393 (23.2)	179 (10.6)	1694	1085 (64.4)	492 (29.2)	109 (6.5)	1686
Female	434 (71.3)	137 (22.5)	38 (6.2)	609	487 (80.0)	105 (17.2)	17 (2.8)	609
Missing/Blank	9	2	0	64	6	4	1	72
P-value				0.004				<.0001
Dentist age in years								
Mean (S.D.)	51.1 (12.3)	50.6 (11.7)	52.8 (10.7)		50.1 (12.2)	52.7 (11.4)	55.2 (10.8)	
P-value				0.086				<.0001
Dentist Hispanic/Latino ethnicity								
Yes	66 (57.9)	30 (26.3)	18 (15.8)	114	72 (63.7)	35 (31.0)	6 (5.3)	113
No	1480 (68.1)	495 (22.8)	198 (9.1)	2173	1490 (68.8)	555 (25.6)	121 (5.6)	2166
Missing/Blank	19	7	1	80	16	11	0	88
P-value				0.026				0.438
Dentist race								
White/Caucasian	1309 (67.9)	436 (22.6)	182 (9.4)	1927	1319 (68.7)	498 (25.9)	104 (5.4)	1921
Black/African-American	75 (67.6)	30 (27.0)	6 (5.4)	111	63 (57.3)	37 (33.6)	10 (9.1)	110
American Indian/Alaska Native	4 (66.7)	1 (16.7)	1 (16.7)	6	6 (100)	0 (0.0)	0 (0.0)	6
Asian	112 (64.7)	44 (25.4)	17 (9.8)	173	123 (71.5)	38 (22.1)	11 (6.4)	172
Native Hawaiian/Pacific Islander	2 (100)	0 (0.0)	0 (0.0)	2	2 (100)	0 (0.0)	0 (0.0)	2
Other	42 (62.7)	15 (22.4)	10 (14.9)	67	43 (64.2)	22 (32.8)	2 (3.0)	67
Missing/Blank	21	6	1	81	22	6	0	89
P-value				0.621				0.163
Year of graduation from dental school								
Mean (S.D.)	1989 (12.7)	1990 (12.2)	1988 (11.2)		1990 (12.6)	1988 (12.2)	1985 (11.4)	
P-value				0.072				<.0001

Characteristic of the dentist, practice, or patient population	Orthodontic treatment None	Occasionally	Routinely	Total		Surgical periodontal therapy None	Occasionally	Routinely	Total
After dental school: No formal training program									
Yes	758 (74.8)	189 (18.7)	66 (6.5)	1013		778 (77.0)	197 (19.5)	35 (3.5)	1010
No	807 (62.0)	343 (26.4)	151 (11.6)	1301		800 (61.7)	404 (31.2)	92 (7.1)	1296
Missing/Blank	0	0	0	53		0	0	0	61
P-value				<.0001					<.0001
After dental school: Completed an AEGD program									
Yes	148 (67.3)	50 (22.7)	22 (10.0)	220		135 (61.6)	71 (32.4)	13 (5.9)	219
No	1417 (67.7)	482 (23.0)	195 (9.3)	2094		1443 (69.1)	530 (25.4)	114 (5.5)	2087
Missing/Blank	0	0	0	53		0	0	0	61
P-value				0.929					0.063
After dental school: Completed a GPR program									
Yes	363 (70.6)	121 (23.5)	30 (5.8)	514		315 (61.9)	159 (31.2)	35 (6.9)	509
No	1202 (66.8)	411 (22.8)	187 (10.4)	1800		1263 (70.3)	442 (24.6)	92 (5.1)	1797
Missing/Blank	0	0	0	53		0	0	0	61
P-value				0.004					0.001
After dental school: I am a FAGD									
Yes	187 (57.9)	94 (29.1)	42 (13.0)	323		177 (55.1)	119 (37.1)	25 (7.8)	321
No	1378 (69.2)	438 (22.0)	175 (8.8)	1991		1401 (70.6)	482 (24.3)	102 (5.1)	1985
Missing/Blank	0	0	0	53		0	0	0	61
P-value				0.0002					<.0001
After dental school: Completed MAGD									
Yes	84 (63.6)	36 (27.3)	12 (9.1)	132		62 (47.0)	57 (43.2)	13 (9.8)	132
No	1481 (67.9)	496 (22.7)	205 (9.4)	2182		1516 (69.7)	544 (25.0)	114 (5.2)	2174
Missing/Blank	0	0	0	53		0	0	0	61
P-value				0.464					<.0001

	Orthodontic treatment					Surgical periodontal therapy			
Characteristic of the dentist, practice, or patient population	None	Occasionally	Routinely	Total		None	Occasionally	Routinely	Total
After dental school: Completed some other training program									
Yes	270 (53.3)	152 (30.0)	85 (16.8)	507		285 (56.7)	172 (34.2)	46 (9.1)	503
No	1295 (71.7)	380 (21.0)	132 (7.3)	1807		1293 (71.7)	429 (23.8)	81 (4.5)	1803
Missing/Blank	0	0	0	53		0	0	0	61
P-value				<.0001					<.0001
Member of: American Dental Association									
Yes	1223 (67.2)	428 (23.5)	169 (9.3)	1820		1227 (67.6)	481 (26.5)	107 (5.9)	1815
No	342 (69.2)	104 (21.1)	48 (9.7)	494		351 (71.5)	120 (24.4)	20 (4.1)	491
Missing/Blank	0	0	0	53		0	0	0	61
P-value				0.502					0.153
Member of: Academy of General Dentistry									
Yes	531 (60.3)	243 (27.6)	107 (12.1)	881		557 (63.5)	266 (30.3)	54 (6.2)	877
No	1034 (72.2)	289 (20.2)	110 (7.7)	1433		1021 (71.4)	335 (23.4)	73 (5.1)	1429
Missing/Blank	0	0	0	53		0	0	0	61
P-value				<.0001					<.0001
Hours in practice in patient contact									
32 or more hours	1259 (65.4)	468 (24.3)	197 (10.2)	1924		1263 (66.0)	537 (28.1)	114 (6.0)	1914
Less than 32 hours	294 (79.2)	61 (16.4)	16 (4.3)	371		304 (81.5)	58 (15.5)	11 (2.9)	373
Missing/Blank	12	3	4	72		11	6	2	80
P-value				<.0001					<.0001
Number of different locations at which you see patients									
1 location	1334 (67.9)	451 (23.0)	179 (9.1)	1964		1361 (69.5)	499 (25.5)	98 (5.0)	1958
2 locations	177 (63.9)	69 (24.9)	31 (11.2)	277		175 (63.6)	78 (28.4)	22 (8.0)	275
3 locations	31 (72.1)	8 (18.6)	4 (9.3)	43		25 (58.1)	13 (30.2)	5 (11.6)	43
More than 3	23 (76.7)	4 (13.3)	3 (10.0)	30		17 (56.7)	11 (36.7)	2 (6.7)	30
Missing/Blank	0	0	0	53		0	0	0	61
P-value				0.644					0.046

	Orthodontic treatment					Surgical periodontal therapy			
Characteristic of the dentist, practice, or patient population	None	Occasionally	Routinely	Total		None	Occasionally	Routinely	Total
Practice location									
Inner City of Urban Area	189 (68.7)	60 (21.8)	26 (9.5)	275		183 (67.5)	71 (26.2)	17 (6.3)	271
Urban (not inner city)	465 (71.9)	133 (20.6)	49 (7.6)	647		449 (69.7)	157 (24.4)	38 (5.9)	644
Suburban	649 (64.2)	265 (26.2)	97 (9.6)	1011		701 (69.4)	254 (25.1)	55 (5.4)	1010
Rural	256 (69.0)	71 (19.1)	44 (11.9)	371		242 (65.2)	113 (30.5)	16 (4.3)	371
Missing/Blank	6	3	1	63		3	6	1	71
P-value				0.010					0.414
Type of main practice (full)									
Owner of private practice	984 (60.0)	459 (28.0)	197 (12.0)	1640		1046 (64.2)	482 (29.6)	102 (6.3)	1630
Associate or employee of a private practice	249 (78.8)	54 (17.1)	13 (4.1)	316		247 (78.4)	56 (17.8)	12 (3.8)	315
HealthPartners Dental Group	49 (96.1)	0 (0.0)	2 (3.9)	51		40 (78.4)	9 (17.6)	2 (3.9)	51
Permanente Dental Associates	62 (98.4)	1 (1.6)	0 (0.0)	63		53 (84.1)	10 (15.9)	0 (0.0)	63
Other managed care or preferred provider organization	14 (82.4)	1 (5.9)	2 (11.8)	17		15 (88.2)	1 (5.9)	1 (5.9)	17
Public health practice, community health center, or publicly-funded clinic (but not a federal facili	112 (96.6)	3 (2.6)	1 (0.9)	116		94 (79.0)	18 (15.1)	7 (5.9)	119
Federal government facility (VA, DoD, Public Health Service)	34 (89.5)	3 (7.9)	1 (2.6)	38		24 (63.2)	13 (34.2)	1 (2.6)	38
Dental school, academic institution, or facility staffed by dental school	57 (86.4)	8 (12.1)	1 (1.5)	66		53 (80.3)	11 (16.7)	2 (3.0)	66
Missing/Blank	4	3	0	60		6	1	0	68
P-value				<.0001					<.0001
Type of main practice (private)									
Private	1358 (65.1)	515 (24.7)	214 (10.3)	2087		1401 (67.5)	558 (26.9)	117 (5.6)	2076
Non private	203 (92.3)	14 (6.4)	3 (1.4)	220		171 (76.7)	42 (18.8)	10 (4.5)	223
Missing/Blank	4	3	0	60		6	1	0	68
P-value				<.0001					0.016
At the same location: Not applicable-only GDs at this location									
Yes	1270 (66.0)	470 (24.4)	185 (9.6)	1925		1277 (66.6)	530 (27.6)	110 (5.7)	1917

	Orthodontic treatment					Surgical periodontal therapy			
Characteristic of the dentist, practice, or patient population	None	Occasionally	Routinely	Total		None	Occasionally	Routinely	Total
No	295 (75.8)	62 (15.9)	32 (8.2)	389		301 (77.4)	71 (18.3)	17 (4.4)	389
Missing/Blank	0	0	0	53		0	0	0	61
P-value				0.0003					0.0001
At the same location: Endodontist									
Yes	88 (80.0)	15 (13.6)	7 (6.4)	110		91 (82.0)	13 (11.7)	7 (6.3)	111
No	1477 (67.0)	517 (23.5)	210 (9.5)	2204		1487 (67.7)	588 (26.8)	120 (5.5)	2195
Missing/Blank	0	0	0	53		0	0	0	61
P-value				0.016					0.001
At the same location: Oral & Maxillofacial Surgeon									
Yes	79 (88.8)	4 (4.5)	6 (6.7)	89		72 (79.1)	14 (15.4)	5 (5.5)	91
No	1486 (66.8)	528 (23.7)	211 (9.5)	2225		1506 (68.0)	587 (26.5)	122 (5.5)	2215
Missing/Blank	0	0	0	53		0	0	0	61
P-value				<.0001					0.047
At the same location: Orthodontist									
Yes	102 (84.3)	12 (9.9)	7 (5.8)	121		97 (78.9)	19 (15.4)	7 (5.7)	123
No	1463 (66.7)	520 (23.7)	210 (9.6)	2193		1481 (67.8)	582 (26.7)	120 (5.5)	2183
Missing/Blank	0	0	0	53		0	0	0	61
P-value				<.0001					0.014
At the same location: Pediatric dentist									
Yes	79 (80.6)	13 (13.3)	6 (6.1)	98		79 (79.8)	16 (16.2)	4 (4.0)	99
No	1486 (67.1)	519 (23.4)	211 (9.5)	2216		1499 (67.9)	585 (26.5)	123 (5.6)	2207
Missing/Blank	0	0	0	53		0	0	0	61
P-value				0.017					0.038
At the same location: Periodontist									
Yes	105 (75.0)	28 (20.0)	7 (5.0)	140		117 (81.8)	22 (15.4)	4 (2.8)	143
No	1460 (67.2)	504 (23.2)	210 (9.7)	2174		1461 (67.5)	579 (26.8)	123 (5.7)	2163
Missing/Blank	0	0	0	53		0	0	0	61

	Orthodontic treatment					Surgical periodontal therapy			
Characteristic of the dentist, practice, or patient population	None	Occasionally	Routinely	Total		None	Occasionally	Routinely	Total
P-value				0.087					0.001
At the same location: Prosthodontist									
Yes	55 (80.9)	11 (16.2)	2 (2.9)	68		51 (72.9)	15 (21.4)	4 (5.7)	70
No	1510 (67.2)	521 (23.2)	215 (9.6)	2246		1527 (68.3)	586 (26.2)	123 (5.5)	2236
Missing/Blank	0	0	0	53		0	0	0	61
P-value				0.040					0.665
At the same location: Other									
Yes	43 (81.1)	5 (9.4)	5 (9.4)	53		33 (64.7)	12 (23.5)	6 (11.8)	51
No	1522 (67.3)	527 (23.3)	212 (9.4)	2261		1545 (68.5)	589 (26.1)	121 (5.4)	2255
Missing/Blank	0	0	0	53		0	0	0	61
P-value				0.042					0.150
How long a patient has to wait: for a new patient exam appt in days									
Mean (S.D.)	8.2 (13.9)	5.5 (6.6)	4.9 (5.9)			7.7 (13.2)	6.6 (9.4)	6.1 (8.9)	
P-value				<.0001					0.094
How long a patient has to wait: for a treatment procedure appt in days									
Mean (S.D.)	8.0 (13.5)	5.4 (6.4)	5.4 (6.3)			7.5 (12.9)	6.9 (9.2)	5.2 (6.3)	
P-value				<.0001					0.089
How long a patient has to wait: in the waiting room in mins									
Mean (S.D.)	8.8 (8.5)	7.6 (6.8)	7.6 (5.8)			8.4 (7.7)	8.4 (8.0)	9.6 (11.1)	
P-value				0.002					0.248
Percentage of patients who are: 1-18 years old									
Mean (S.D.)	18.3 (16.6)	15.4 (11.2)	19.3 (16.1)			18.4 (16.7)	16.2 (12.5)	16.2 (11.8)	
P-value				0.0004					0.006

	Orthodontic treatment					Surgical periodontal therapy			
Characteristic of the dentist, practice, or patient population	None	Occasionally	Routinely	Total		None	Occasionally	Routinely	Total
Percentage of patients who are: 19-44 years old									
Mean (S.D.)	28.6 (12.7)	29.7 (12.0)	29.2 (11.9)			28.9 (12.7)	29.0 (12.2)	28.1 (10.6)	
P-value				0.193					0.760
Percentage of patients who are: 45-64 years old									
Mean (S.D.)	32.8 (12.5)	35.0 (10.7)	32.7 (12.1)			32.9 (12.2)	34.2 (11.6)	34.7 (13.3)	
P-value				0.001					0.027
Percentage of patients who are: 65 or older									
Mean (S.D.)	20.3 (12.1)	19.9 (10.0)	18.8 (11.3)			19.8 (11.6)	20.6 (11.4)	21.0 (12.2)	
P-value				0.154					0.239
Percentage of patients who are of Hispanic/Latino ethnicity									
Mean (S.D.)	15.2 (19.6)	13.2 (15.4)	19.6 (22.7)			15.1 (19.7)	15.7 (18.1)	14.0 (17.2)	
P-value				0.0003					0.637
Percentage of patients whose race is: White/Caucasian									
Mean (S.D.)	67.9 (24.1)	70.6 (21.3)	69.3 (22.0)			68.8 (23.3)	68.5 (23.4)	67.4 (22.9)	
P-value				0.067					0.824
Percentage of patients whose race is: Black/African-American									
Mean (S.D.)	17.5 (17.6)	15.7 (16.2)	14.8 (14.9)			16.5 (16.8)	17.3 (17.6)	18.7 (17.7)	
P-value				0.024					0.291
Percentage of patients whose race is: American Indian/Alaska Native									
Mean (S.D.)	1.8 (7.9)	1.2 (3.6)	1.9 (7.1)			1.5 (6.3)	2.1 (8.4)	1.6 (8.5)	
P-value				0.294					0.274

	Orthodontic treatment					Surgical periodontal therapy			
Characteristic of the dentist, practice, or patient population	None	Occasionally	Routinely	Total		None	Occasionally	Routinely	Total
Percentage of patients whose race is: Asian									
Mean (S.D.)	6.2 (7.2)	6.8 (8.6)	6.2 (7.6)			6.4 (7.7)	6.3 (7.6)	6.1 (6.6)	
P-value				0.272					0.926
Percentage of patients whose race is: Native Hawaiian/Pacific Islander									
Mean (S.D.)	0.7 (2.3)	0.9 (2.5)	0.8 (2.1)			0.7 (2.1)	1.0 (2.7)	0.8 (2.6)	
P-value				0.600					0.053
Percentage of patients whose race is: Other									
Mean (S.D.)	5.9 (15.8)	4.8 (12.3)	7.0 (13.8)			6.1 (16.0)	4.9 (11.9)	5.4 (13.8)	
P-value				0.137					0.200
Percentage of patients: Covered by a private insurance program									
Mean (S.D.)	57.3 (26.9)	62.8 (20.5)	59.6 (20.7)			59.1 (26.0)	58.3 (23.6)	56.5 (21.5)	
P-value				<.0001					0.502
Percentage of patients: Covered by a public program									
Mean (S.D.)	16.6 (25.8)	8.7 (17.7)	10.1 (17.8)			15.1 (24.4)	12.6 (22.5)	10.9 (20.6)	
P-value				<.0001					0.030
Percentage of patients: Not covered by any third party and pays out of pocket									
Mean (S.D.)	21.5 (17.8)	26.0 (16.7)	27.3 (16.8)			21.8 (17.7)	25.1 (17.0)	29.4 (17.6)	
P-value				<.0001					<.0001
Percent of patients: Receiving free care or substantially reduced fees courtesy of this practice									
Mean (S.D.)	4.4 (12.3)	2.5 (4.0)	3.0 (4.6)			3.9 (10.7)	3.9 (10.1)	3.2 (9.5)	
P-value				0.001					0.755

	Orthodontic treatment					Surgical periodontal therapy			
Characteristic of the dentist, practice, or patient population	None	Occasionally	Routinely	Total		None	Occasionally	Routinely	Total
Percent of patients who come: For one visit only									
Mean (S.D.)	8.9 (10.9)	7.4 (6.9)	9.1 (9.0)			8.8 (10.6)	8.2 (8.7)	8.0 (8.3)	
P-value				0.008					0.337
Percent of patients who come: Occasionally only when they have an emergency or specific problem									
Mean (S.D.)	13.3 (9.8)	12.3 (8.9)	12.6 (9.5)			13.1 (10.0)	13.0 (8.7)	12.7 (8.8)	
P-value				0.078					0.876
Percent of patients who come: Irregularly whether or not they have a problem									
Mean (S.D.)	16.4 (10.9)	15.8 (9.2)	15.6 (10.3)			15.9 (10.2)	16.9 (10.6)	16.5 (12.3)	
P-value				0.339					0.166
Percent of patients who come: Regularly as recommended or whether or not they have a problem									
Mean (S.D.)	61.3 (20.4)	64.5 (17.2)	62.7 (19.2)			62.1 (19.8)	62.0 (19.1)	62.8 (20.3)	
P-value				0.005					0.914

[a] This table is limited to the 2,367 GDs who reported their generalist/specialist status. This includes enrollments as of October 31, 2013.

[b] P-values for the association between the characteristic and the procedure computed using Fisher's exact test (for categorical variables) and analysis of variance (for numerical variables).

GD: general dentist
AEGD: Advanced Education in General Dentistry
GPR: General Practice Residency
FAGD: Fellow of the Academy of General Dentistry
MAGD: Master of the Academy of General Dentistry
